# What do we know about managing Dupuytren’s disease cost-effectively?

**DOI:** 10.1186/s12891-018-1949-2

**Published:** 2018-01-25

**Authors:** Melina Dritsaki, Oliver Rivero-Arias, Alastair Gray, Catherine Ball, Jagdeep Nanchahal

**Affiliations:** 1 Oxford Clinical Trials Research Unit, Nuffield Department of Orthopaedics, Rheumatology and Musculoskeletal Sciences, University of Oxford, Oxford, OX3 7LD UK; 2 National Perinatal Epidemiology Unit (NPEU), Nuffield Department of Population Health, University of Oxford, Oxford, OX3 7LF UK; 3 Health Economics Research Centre, Nuffield Department of Population Health, University of Oxford, Oxford, OX3 7LF UK; 4 Kennedy Institute of Rheumatology, Nuffield Department of Orthopaedics, Rheumatology and Musculoskeletal Sciences, University of Oxford, Oxford, OX3 7FY UK

**Keywords:** Dupuytren’s disease, Systematic review, Economic evaluation, Economic modelling

## Abstract

**Background:**

Dupuytren’s disease (DD) is a common and progressive, fibroproliferative disorder of the palmar and digital fascia of the hand. Various treatments have been recommended for advanced disease or to retard progression of early disease and to prevent deterioration of the finger contracture and quality of life. Recent studies have tried to evaluate the clinical and cost-effectiveness of therapies for DD, but there is currently no systematic assessment and appraisal of the economic evaluations.

**Methods:**

A systematic literature review was conducted, following PRISMA guidelines, to identify studies reporting economic evaluations of interventions for managing DD. Databases searched included the Ovid MEDLINE/Embase (without time restriction), National Health Service (NHS) Economic Evaluation Database (all years) and the National Institute for Health Research (NIHR) Journals Library) Health Technology Assessment (HTA). Cost-effectiveness analyses of treating DD were identified and their quality was assessed using the CHEERS assessment tool for quality of reporting and Phillips checklist for model evaluation.

**Results:**

A total of 103 studies were screened, of which 4 met the study inclusion criteria. Two studies were from the US, one from the UK and one from Canada. They all assessed the same interventions for advanced DD, namely collagenase Clostridium histolyticum injection, percutaneous needle fasciotomy and partial fasciectomy. All studies conducting a cost-utility analysis, two implemented a decision analytic model and two a Markov model approach. None of them were based on a single randomised controlled trial, but rather synthesised evidence from various sources. Studies varied in their time horizon, sources of utility estimates and perspective of analysis. The overall quality of study reporting was good based on the CHEERS checklist. The quality of the model reporting in terms of model structure, data synthesis and model consistency varied across the included studies.

**Conclusion:**

Cost-effectiveness analyses for patients with advanced DD are limited and have applied different approaches with respect to modelling. Future studies should improve the way they are conducted and report their findings according to established guidance for conducting economic modelling of health care technologies.

**Trial registration:**

The protocol was registered (CRD42016032989; date 08/01/2016) with the PROSPERO international prospective register of systematic reviews.

**Electronic supplementary material:**

The online version of this article (10.1186/s12891-018-1949-2) contains supplementary material, which is available to authorized users.

## Background

Dupuytren’s disease (DD) affects 4% of the general UK population and is a progressive, fibro-proliferative condition affecting the palmar and digital fascia of the hand, the ‘bands’ that anchor the skin of the palm [1–3]. Early manifestation of the disease is as a firm nodule. These nodules are the precursors to the development of fibrous collagenous cords which extend into the fingers. With further disease progression, cords thicken and contract, causing finger(s) to curl irreversibly into the palm. Approximately 40% of patients with early disease might eventually be expected to progress to develop cords [4, 5] and the flexion deformities that impair hand function, thereby greatly limiting activities of daily life, including self-care, usual activities and employment, and reducing health-related quality of life (HRQoL) [6].

The number of patients requiring treatment for DD in England increased by almost 50% between 1998 and 2011 (from 11,716 to 17,342 per annum). With the aging population it is predicted that there will be a further increase in the numbers of patients requiring treatment through to 2030 [7]. In an era of scarce health care resources, competing health care interventions must be subject to assessment of cost-effectiveness to identify those offering best value for money. Treatments for DD are no exception, and ensuring the condition is managed in a cost-effective way is especially important given the increasing demand on services placed by patients with the condition in the future.

Treatment for DD is recommended when the digital flexion contractures limit hand function and/or the proximal interphalangeal joint is flexed to 30^0^ or more [3] and aims to correct the flexion deformities and restore hand function. Surgical interventions include fasciectomy whereby the diseased cords are surgically excised, usually under general or regional anaesthesia, or fasciotomy, where the cords are divided using a needle [8]. More recently there has been interest in using collagenase from Clostridium histolyticum, an enzyme which breaks down and weakens the collagen in the cords. Collagenase is injected directly into the affected tissue and manual straightening of the finger is performed 24 to 72 h later [9, 10]. Trade-offs are likely to exist when comparing the three treatments in terms of procedure-related morbidity and complications (likely to be higher with the more invasive fasciectomy), treatment failure and recurrence (potentially higher with fasciotomy and collagenase which both leave residual diseased tissue), time to return to work (likely to be longer with the more invasive fasciectomy), and cost [11, 12].

Treatments to retard the progression of early disease and to prevent both the deterioration in HRQoL associated with finger contracture and necessity for intervention and disease recurrence, have also been researched. A recent systematic review identified studies evaluating the clinical outcomes of radiotherapy, steroids, and physical therapy in patients with early DD [13]. A trial to evaluate the efficacy of a drug to inhibit tumour necrosis factor is ongoing (Repurposing Anti-TNF for Treating Dupuytren’s Disease (RIDD) trial (ISRCTN27786905 DOI 10.1186/ISRCTN27786905) based on laboratory data showing the key role of this cytokine in the development and maintenance of the phenotype of myofibroblasts [14], the cells responsible for both the excessive matrix deposition and contraction [15].

The aim of this study is to conduct a systematic literature review to identify the extent and appraise the quality of the existing literature on the cost-effectiveness of treatments for DD.

## Methods

The search included Ovid Medline/Embase (without time restriction), the NHS Economic Evaluation Database (NHS EED available at http://www.crd.york.ac.uk/CRDWeb/) (all years) and Health Technology Assessment (HTA) (available at http://www.crd.york.ac.uk/CRDWeb/). Following extraction of eligible papers, forward and back-citation searches were conducted using Google Scholar. When potentially eligible abstracts were identified, the authors were contacted to ascertain whether further details of the study were available. The search strategy for Ovid Medline is shown in Additional file 1 and contains a combination of MeSH headings and free text words relating to both the clinical condition and health economic evaluation. We used the search strategy originally developed by Brazzelli et al. [2] on the cost-effectiveness of interventions for DD, which has proven to be sensitive and specific, with the inclusion of an additional clinical term (see line 4 in Additional file 1).

The search aimed to identify any study reporting on the cost-effectiveness of one or more interventions for managing DD in adult patients aged 18 years and over. Invasive and non-invasive interventions for both early and advanced disease were sought, as were all types of full economic evaluation, including cost-consequence analysis (CCA), cost-effectiveness analysis (CEA), cost-utility analysis (CUA), and cost-benefit analysis (CBA). Any study design was permitted, including economic evaluations conducted as part of patient-based studies such as randomised controlled trials, non-randomised studies and decision analytical models. To identify current practices, only studies published between January 1996 and November 2016 were eligible for inclusion. No restrictions were placed on language or on publication type. Studies reporting on the management of DD prior to 1996, or in adolescents, were excluded, as were cost minimisation analyses and cost analyses whereby interventions are compared on the basis of cost only. The review was registered on the PROSPERO register of systematic reviews (registration number CRD42016032989).

All records returned by searching were imported into EndNote bibliographic software (EndNote 2016 Thomson Reuters) and duplicate publications removed. A first screen of titles and abstracts was conducted by one reviewer (OR-A) and full text versions of potentially eligible publications were obtained. Two reviewers (MD and OR-A) then independently read and classified each full text publication as eligible or not based upon the criteria outlined above. The resulting classifications were compared and disagreements resolved through discussion.

The Consolidated Health Economic Evaluation Reporting Standards (CHEERS) statement [16] was used to extract data and also assess the quality of data reporting (see Additional file 2). CHEERS was developed using CONSORT methodology [17] to improve the quality of the reporting of economic evaluations and includes a checklist of 24 items. The items include study title, background and objectives, target population, setting and location, treatment alternatives being compared, methodological issues such as study time horizon, use of discount rates for both costs and outcomes, choice of models applied, assumptions made, and ways of accounting for model uncertainty and heterogeneity. Economic evaluations based on a single study and those based on evidence synthesis from a variety of sources were also distinguished. Two authors (MD and OR-A) independently extracted data from the included studies, and any disagreements were again resolved through discussion. We contacted the authors of three published abstracts for further information. All three responded and two were able to provide further information in the form of conference poster presentations.

A literature review reported existing reporting guidelines for economic evaluations that were relevant to population modelling studies [18]. The authors identified a total of 69 quality criteria among seven economic evaluation studies, including one which provided a detailed framework for model quality assessment [19] (see Additional file 3). The overarching themes of the checklist published by these authors relate to 1) structure of the model (20 items), 2) data issues (31 items), 3) consistency (& validity) of the model (5 items). Two authors (MD and OR-A) independently assessed the models from the included studies against the Philips checklist [19]. The purpose of this exercise was to identify, in a systematic approach, the strengths and any particular issues or weakness of the models identified so as to inform any future modelling applications.

## Results

### Summary of studies included in the review

**Fig. 1:**
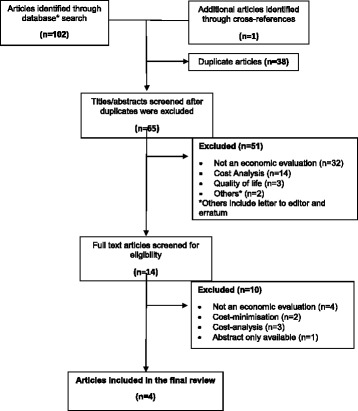
PRISMA flowchart of inclusion and exclusion of articles incorporated in the review. *Databases included Ovid Medline, Ovid Embase and NHS EED

The searches of Ovid Medline, Embase and NHS EED identified 102 studies, and one additional publication was included from cross-references. Thirty-eight duplicate articles were identified and removed, leaving the titles and abstracts of 65 publications to be screened (by OR-A and MD) (see Additional file 4 for details). Fifty-one of these publications were not eligible for inclusion in the review: 32 did not include an economic evaluation, 14 reported a cost-analysis only, 3 conducted a quality of life analysis only, and 2 manuscripts were a letter to the editor and an erratum. The remaining 14 publications were obtained in full, and following independent review and discussion, four were considered eligible for inclusion [2, 20–22], two of which overlap with the findings reported by Brazzelli et al. [2]. Figure 1 (PRISMA flowchart) reports a breakdown of the reasons for exclusion of the remaining 10 manuscripts at this stage.

A search of the HTA database returned ten potentially eligible records that were studied for additional missing references. Five records and their associated publications were reviewed but revealed no further eligible studies. The remaining five were for commercially conducted HTAs covering two interventions for advanced DD. The reports could be purchased but no summary information was available about the content with regards to cost-effectiveness analysis and therefore they were excluded.

Details on the CHEERS assessment of the four included studies are described in Additional file 5. None of the studies evaluated the cost-effectiveness of interventions for early DD. Published from 2011 onwards, all four (two from the US, one from Canada, and one from the UK) assessed the same three interventions for patients with advanced disease manifest as contractures of one or more digits, namely collagenase Clostridium histolyticum, percutaneous needle fasciotomy (aponeurotomy) and partial (limited) fasciotomy. Two were full journal articles, one a chapter in a Health Technology Assessment (HTA) report, and one was a conference poster presentation. All four utilised modelling as the framework for estimating costs and effects.

Summary conclusions differed across the four studies (see Additional file 5). Two concluded that fasciectomy was not cost-effective, and that collagenase was unlikely to offer value for money unless the injection cost was significantly lower than the current market price [20, 22]. In contrast, the third report found fasciectomy to be the most cost-effective treatment, dominating both collagenase and percutaneous needle fasciotomy [21]. The fourth study also concluded that fasciectomy offered the best value for money, dominating collagenase and offering a favourable cost per QALY when compared with percutaneous fasciotomy [2].

### Quality of reporting

Table 1 summarises the CHEERS assessment results of the four studies included in the current systematic literature review. The overall quality of the papers was good. All the studies conducted a cost-utility analysis. Two [20, 22] implemented an expected value decision analytic model whereas the others [2, 21] employed a Markov model. None of the studies was based on a single RCT, but rather used evidence synthesised from various sources. The time horizon varied between the studies, ranging from 10 to 37 years, with a starting age of 63 years assumed in all studies apart from one [21] which assumed a starting age of 50. All the studies discounted costs and effects at 3%, 3.5% or 5% except one [20], which did not report discounting for future cost and outcomes, although the time horizon of the analysis was more than 1 year. Uncertainty was addressed in 3 out of 4 studies, but only one group [2] conducted a probabilistic sensitivity analysis to test the robustness of results to simultaneous changes in model parameters. Characterisation of both deterministic and probabilistic sensitivity analysis is particularly important in decision analytical model and therefore should be included [23].

**Table 1:** Reporting standards in the included studies

		Yes	No	Not Applicable
CHEERS reporting item				
1	Title	4		
2	Abstract	4		
3	Background and objectives	4		
4	Target population and subgroups	4		
5	Setting and location	4		
6	Study perspective	4		
7	Comparators	4		
8	Time horizon	4		
9	Discount rate	3	1	
10	Choice of health outcomes	4		
11a	Measurement of effectiveness (single study-based estimates)			4
11b	Measurement of effectiveness (synthesis-based estimates)	4		
12	Measurement and valuation of preference-based outcomes	4		
13a	Estimating resources and costs (single study-based economic evaluation)			4
13b	Estimating resources and costs (model-based economic evaluation)	4		
14	Currency, price date and conversion	4		
15	Choice of model	4		
16	Assumptions	4		
17	Analytic method	4		
18	Study parameters	4		
19	Incremental costs and outcomes	3	1	
20a	Characterising uncertainty (single study-based economic evaluation)			4
20b	Characterising uncertainty (model-based economic evaluation)	3	1	
21	Characterising heterogeneity	1	3	
22	Study findings, limitations, generalisability and current knowledge	4		
23	Source of funding		3	1
24	Conflicts of interest		3	1

Sources of utility estimates varied across the four included studies: two studies [20, 22] conducted a utility survey using standard gamble techniques (SG) specific to their study in a US setting and surveyed participants living with Dupuytren’s contracture. One study [21] used utility valuations from patients with carpal tunnel syndrome, and another one [2] used a discrete choice experiment (DCE) based on the general UK population [24]. For the latter, the values were scaled to be approximately relative to an EQ-5D-3 L health state. QALYs derived from the US survey-SG questionnaire ranged from 0.971 for successful treatment with complications to 0.994 for successful treatment without any complications. When no treatment was provided, the utility was 0.987. The DCE approach provided results that were significantly different to the SG-based results, with utilities for affected dominant hands, non-dominant hands or ambidextrous hands being respectively 0.49, 0.57 and 0.63 [24].

The framework described by Phillips et al. [19] for quality assessment was applied to evaluate the models of the four included studies (see Additional file 6). Table 2 summarises the evidence in terms of model structure, data synthesis and model consistency. All authors provided a clear statement about the decision problem, prompting the analysis which in all cases defined the population as Duyputren’s contracture and the available treatment options. The perspective of the analyses, although defined clearly, was not always consistent with the scope of the models. For example, although one study considered a societal perspective, the authors did not include any cost bourne by patients, out of pocket expenses or productivity loss due to treatment for Dupuytren’s contracture [20]. All treatment comparators evaluated within the models were feasible and practical within the reference healthcare systems. All studies compared up to three treatment options and none of them provided justification of the exclusions of any other feasible options. Vehicle to calculate the decision analysis were decision trees and Markov models. Although all models took a long term or life time horizon, only two publications [2, 22] provided justification of the time frame used.

**Table 2:** Reporting standards for modelling studies [19]

Quality criteria	Question(s) for critical appraisal	Yes	No	YES/NO	?	Not applicable
Structure (S)						
S1	Is there a clear statement of the decision problem?	4				
	Is the objective of the evaluation and model specified and consistent with the stated decision problem?	4				
	Is the primary decision maker specified?		4			
S2	Is the perspective of the model stated clearly?	4				
	Are the model inputs consistent with the stated perspective?	2	1		1	
	Has the scope of the model been stated and justified?	2		2		
	Are the outcomes of the model consistent with the perspective, scope and overall objective of the model?	3	1			
S3	Has the evidence regarding the model structure been described? Is the structure of the model consistent with a coherent theory of the health condition under evaluation?	4				
	Are the sources of data used to develop the structure of the model specified?	4				
	Are the causal relationships described by the model structure justified appropriately?		4			
S4	Are the structural assumptions transparent and justified?	3		1		
	Are the structural assumptions reasonable given the overall objective, perspective and scope of the model?	4				
S5	Is there a clear definition of the options under evaluation?	4				
	Have all feasible and practical options been evaluated?		4			
	Is there justification for the exclusion of feasible options?		4			
S6	Is the chosen model type appropriate given the decision problem and specified causal relationships within the model?	3		1		
S7	Is the time horizon of the model sufficient to reflect all important differences between options?	3			1	
	Is the time horizon of the model, the duration of treatment and the duration of treatment effect described and justified?	2	1	1		
S8	Do the disease states (state transition model) or the pathways (decision tree model) reflect the underlying biological process of the disease in question and the impact of interventions?	4				
S9	Is the cycle length defined and justified in terms of the natural history of disease?	1		1		2
DATA (D)						
D1	Are the data identification methods transparent and appropriate given the objectives of the model?	4				
	Where choices have been made between data sources, are these justified appropriately?	3				1
	Has particular attention been paid to identifying data for the important parameters in the model?	4				
	Has the process of selecting key parameters been justified and systematic methods used to identify the most appropriate data?	3	1			
	Has the quality of the data been assessed appropriately?		4			
	Where expert opinion has been used, are the methods described and justified?	1				3
D2	Is the pre-model data analysis methodology based on justifiable statistical and epidemiological techniques?		3		1	
D2a	Is the choice of baseline data described and justified?	2	2			
	Are transition probabilities calculated appropriately?	2				2
	Has a half cycle correction been applied to both cost and outcome?	1		1		2
	If not, has this omission been justified?		1			3
D2b	If relative treatment effects have been derived from trial data, have they been synthesised using appropriate techniques?		1			3
	Have the methods and assumptions used to extrapolate short-term results to final outcomes been documented and justified?	2	1	1		
	Have alternative extrapolation assumptions been explored through sensitivity analysis?	2	2			
	Have assumptions regarding the continuing effect of treatment once treatment is complete been documented and justified?	3	1			
	Have alternative assumptions regarding the continuing effect of treatment been explored through sensitivity analysis?	1	3			
D2c	Are the utilities incorporated into the model appropriate?			1	3	
	Is the source for the utility weights referenced?	3	1			
	Are the methods of derivation for the utility weights justified?	2	2			
D3	Have all data incorporated into the model been described and referenced in sufficient detail?	3		1		
	Has the use of mutually inconsistent data been justified (i.e. are assumptions and choices unclear appropriate)?					4
	Is the process of data incorporation transparent?	3		1		
	If data have been incorporated as distributions, has the choice of distribution for each parameter been described and justified?			2		2
	If data have been incorporated as distributions, is It clear that second order uncertainty is reflected?		2			2
D4	Have the four principal types of uncertainty been addressed?	1	3			
	If not, has the omission of particular forms of uncertainty been justified?		3			1
D4a	Have methodological uncertainties been addressed by running alternative versions of the model with different methodological assumptions?	1	3			
D4b	Is there evidence that structural uncertainties have been addressed via sensitivity analysis?	1	3			
D4c	Has heterogeneity been dealt with by running model separately for different sub-groups?	1	3			
D4d	Are the methods of assessment of parameter uncertainty appropriate?	4				
	If data are incorporated as point estimates, the ranges used for sensitivity analysis stated clearly and justified?	2		1		1
Consistency (C)						
C1	Is there evidence that the mathematical logic of the model has been tested thoroughly before use?	2	2			
C2	Are the conclusions valid given the data presented?	4				
	Are any counterintuitive results from the model explained and justified?	2				2
	If the model has been calibrated against independent data, have any differences been explained and justified?	1				3
	Have the results of the model been compared with those of previous models and any differences in results explained?	2	2			

Methods used to identify data were transparent in all studies and consistent with the objectives of the models. The quality and reliability of the retrieved data and data input parameters were not described in any of the studies, although trial data or prospective naturalistic studies are recommended as the highest quality sources of data [25]. An example of a parameter that may have a significant impact on the modelling process for treatments Dupuytren’s disease is the definition of risk of recurrence. Further treatment option following a treatment failure should be based on clinical opinion and expertise. Also, decisions should be made on whether patients failing after a specific treatment should proceed for further treatment or instead enter a semi-absorbing state with no accumulated cost and lower health related quality of life.

Data parameters were incorporated in the studies either as point estimates (deterministic) or as distributions (probabilistic), or both [2]. In the first case, parameter uncertainty was explored through univariate sensitivity analysis on recurrence rate, complication rate and various cost items [20–22]. In the case of probabilistic parameter distributions the functional form of the distributions was described but not justified clearly. Only one study [2] made a distinction and addressed different forms of uncertainty, namely parameter, structural and methodological. Only two studies [2, 22] examined the external consistency of their models by comparing their results with available evidence and other models that addressed similar research questions. The internal consistency (mathematical logic) of the model was partly assessed in the two aforementioned studies as well.

## Discussion

In this study we searched for and systematically reviewed cost-effectiveness analyses of potential interventions for DD. We identified only four studies (3 peer-review manuscripts and one poster conference presentation), suggesting that the country-specific economic evidence to date is sparse. All four studies used an economic model and focused on late rather than early stage DD. The quality of reporting varied, but overall was good. Studies used different time horizons and had different analytical perspectives and different pricing systems. These differences had a major impact on the cost effectiveness results, with two studies [20, 22] suggesting that fasciectomy was not cost-effective whereas the remaining two studies [2, 21] concluded that fasciectomy was the most cost-effective treatment. None of the studies used a common guideline to report the results of their modelling exercise. Therefore, it is difficult to assess how the results of a study in a particular country are relevant to other jurisdictions.

The quality-adjusted life year (QALY) was the main health outcome measure in all models. Two main approaches were taken to derive utility estimates: the standard gamble (SG) approach, and discrete choice experiments (DCEs). The standard gamble approach is associated with a number of limitations [2, 24]. In particular, participants offered a choice between two extreme alternative outcomes, such as taking a pill (which would lead to an immediate state of full health or immediate death), or living in a particular chronic state for the rest of their life, were often strongly death-averse, with the result that very high utilities (0.971 to 0.994) were estimated for some health states. The resulting implication is that for an intervention to be cost-effective it should be both highly successful and very inexpensive [20], which suggests that the utility elicitation method is unrealistic and lacks face validity [20].

The authors of the included studies primarily used decision modelling to estimate costs and outcomes of alternative treatments for DD. The versatility of health economic modelling was likely to be well suited to the assessment of cost-effectiveness in the field of DD for a number of reasons. Firstly, treatments span both early and advanced disease, and modelling can facilitate a comparison of early versus late intervention in the absence of trial-based head to head comparisons. Secondly, progression and recurrence of DD following initial treatment is common, and modelling can be used to extrapolate and capture the costs and consequences of these repeated events into the future. Thirdly, modelling can be used to identify future research priorities in the field.

A potential limitation of the paper is that it cannot be considered as a meta-analysis of the cost-effectiveness of treatment of Dupuytren’s disease, and does not use statistical techniques to combine the results from retrieved studies to obtain a quantitative estimate of the effect of a particular intervention or outcome. The aim of this study was to conduct a systematic literature review to identify any existing literature on the cost-effectiveness of treatments for DD and assess the quality of such studies. We are not using any primary data because this literature review will ultimately inform the economic evaluation and modelling alongside a pragmatic randomised clinical trial (RIDD). Another limitation arises from the potential underestimation of the quality of the model applied to the publication by Sau et al. [21] based on the Phillips checklist. The nature of evidence (provided in a conference abstract format) makes it difficult to fully critically appraise it since available information is restricted to a page. Regardless of this limitation, the inclusion of this publication in this literature review was considered essential.

Recommendations for the design of a model- based economic evaluation of Dupuytren’s disease:

- We recommend that an improved and systematic reporting following CHEERS standards to facilitate interpretation and comparison between studies and help to clearly identify study methods, quality and limitations. If possible, both a healthcare and broader societal perspective should be adopted. DD affects daily function as well as capacity to work (productivity loss), and these wider effects should also be captured. Employment-related information should be collected using a custom-made questionnaire specific for this patient population.
- DD is a musculoskeletal disease which, although not life-threatening, has a chronic element. Therefore, a lifetime perspective should be taken when modelling costs and outcomes beyond a trial’s time horizon. Costs and outcomes should be discounted at the customary 3.5% discount rate.
- Quality-adjusted life years (QALYs) should be used as the main health outcome measure in the model.
- The structure of the model and the identification of the Dupuytren’s disease health state history should be developed using previous published epidemiological and economic models and in discussion with clinicians treating patients with the disorder. Transition probabilities associated with each health state should be taken from patient-level data and/or from the literature. When the disease recurs or is predicted to recur, the impact of any associated surgical treatment on costs and quality of life should be evaluated. Information on rates of recurrence and their impact on costs and quality of life should be taken from the literature.
- The model structure, the uncertainty around data parameters, and the internal and external validation of the model should receive particular attention during the preparation of the model. Uncertainty around cost-effectiveness results should be handled using probabilistic sensitivity analysis and cost-effectiveness acceptability curves. Areas of methodological uncertainty, such as discount rates for costs and outcomes, should be explored using sensitivity analysis. Heterogeneity should be explored using pre-specified sub-groups. Current value judgements from the National Institute for Health and Clinical Excellence should be used to guide willingness to pay for health benefits and hence calculate likelihood of being cost-effective [26].

We anticipate the above recommendation to contribute to the planned economic analysis alongside the Repurposing Anti-TNF for Treating Dupuytren’s Disease (RIDD) trial (ISRCTN27786905 DOI 10.1186/ISRCTN27786905), which began recruiting patients in 2016. Identification of previous cost-effectiveness analyses will help identify likely cost drivers, key model parameters and variables most likely to require sensitivity analyses, and provide context for new evidence. The economic analysis alongside the RIDD study is expected to synthesise information from the trial with literature searches to populate a model evaluating alternative treatments to delay the progression to advanced DD. The model will capture progression and recurrence of DD following initial treatment and will extrapolate and capture the future costs and consequences of these repeated events. It will also help identify and prioritise future research priorities in this field.

## Conclusion

Our results suggest that the current cost-effectiveness evidence on potential treatments for patients with DD is limited. We have identified areas for improvement in the reporting and conduct of these studies, in particular with respect to modelling. The conduct and reporting of future studies evaluating the cost-effectiveness of alternative treatments for DD should be improved. This can be achieved using established guidance on good research practice for the conduct of economic modelling of health care technologies [27].

## Additional files

Additional file 1: Search strategy used in the systematic review. (DOCX 12 kb)

Additional file 2: CHEERS checklist-item to include when reporting economic evaluations of health interventions [16]. (DOCX 18 kb)

Additional file 3: Checklist items for reporting modelling studies [19]. (DOCX 18 kb)

Additional file 4: List of 65 publications identified in the first stage after removing duplicates. (DOCX 23 kb)

Additional file 5: Cheers Assessments of included studies. (DOCX 36 kb)

Additional file 6: Quality assessments framework to the modelling studies [19]. (DOCX 40 kb)

## Abbreviations

CBA: Cost-benefit analysis
CCA: Cost-consequence analysis
CEA: Cost-effectiveness analysis
CHEERS: Consolidated health economics evaluation reporting standards
CUA: Cost-utility analysis
DCE: Discrete choice experiment
DD: Dupuytren’s Disease
HRQoL: Health- Related Quality of Life
HTA: Health technology assessment
NHS: National Healthcare System
QALY: Quality adjusted life years
RCT: Randomised controlled trial
SG: Standard and gamble
