# Supplementary material for: What do we know about managing Dupuytren’s disease cost-effectively?
Source: BMC Musculoskelet Disord. 2018 Jan 25;19:34. doi: 10.1186/s12891-018-1949-2 (PMC5785840; doi:10.1186/s12891-018-1949-2)
Supplement: Supplementary file 1 — Search strategy used in the systematic review. (DOCX 12 kb) [file 12891_2018_1949_MOESM1_ESM.docx]

**Additional file 1**: Search strategy used in the systematic review

| **Set** | **Search Statement** |
| --- | --- |
| 1 | Dupuytren Contracture.mp. or Dupuytren Contracture/ |
| 2 | dupuytren$.tw. |
| 3 | (palm$ adj3 fibromatosis).tw. |
| 4 | ((palm$ adj3 fascia$) and contract$).mp. |
| 5 | 1 or 2 or 3 or 4 |
| 6 | costs.mp. or "Costs and Cost Analysis"/ |
| 7 | economic evaluation.mp. or Cost-Benefit Analysis/ |
| 8 | Economics, Pharmaceutical/ or Economics, Medical/ or Economics/ or Economics, Hospital/ |
| 9 | Cost-Benefit Analysis/ or health economics.mp. or Health Services Research/ |
| 10 | budgets.mp. or Budgets/ |
| 11 | Models, Economic/ |
| 12 | Decision Theory/ |
| 13 | Monte Carlo Method/ |
| 14 | Markov Chains/ |
| 15 | Technology Assessment, Biomedical/ec [Economics] |
| 16 | cost$.ti. |
| 17 | (cost$ adj2 (effective$ or utilit$ or benefit$ or minimis$)).ab. |
| 18 | economics model$.tw. |
| 19 | (economic$ or pharmacoeconomic$).tw. |
| 20 | (price or prices or pricing).tw. |
| 21 | (value adj1 money).tw. |
| 22 | markov$.tw. |
| 23 | monte carlo.tw. |
| 24 | (decision$ adj2 (tree? or analy$ or model$)).tw. |
| 25 | 6 or 7 or 8 or 9 or 10 or 11 or 12 or 13 or 14 or 15 or 16 or 17 or 18 or 19 or 20 or 21 or 22 or 23 or 24 |
| 26 | 5 and 25 |
