# Supplementary material for: What do we know about managing Dupuytren’s disease cost-effectively?
Source: BMC Musculoskelet Disord. 2018 Jan 25;19:34. doi: 10.1186/s12891-018-1949-2 (PMC5785840; doi:10.1186/s12891-018-1949-2)
Supplement: Supplementary file 3 — Checklist items for reporting modelling studies [19]. (DOCX 18 kb) [file 12891_2018_1949_MOESM3_ESM.docx]

**Additional file 3**: **Checklist items for reporting modelling studies [19]**

| **Quality**  **criteria** | **Question(s) for critical appraisal** | **Response** | **Comments** |
| --- | --- | --- | --- |
| **Structure (S)** | | | |
| **S1** | Is there a clear statement of the decision problem? |  |  |
|  | Is the objective of the evaluation and model specified and consistent with the stated decision  problem? |  |  |
|  | Is the primary decision maker specified? |  |  |
| **S2** | Is the perspective of the model stated clearly? |  |  |
|  | Are the model inputs consistent with the stated  perspective? |  |  |
|  | Has the scope of the model been stated and justified? |  |  |
|  | Are the outcomes of the model consistent with  the perspective, scope and overall objective of  the model? |  |  |
| **S3** | Has the evidence regarding the model structure been described?  Is the structure of the model consistent with a coherent theory of the health condition under evaluation? |  |  |
|  | Are the sources of data used to develop the structure of the model specified? |  |  |
|  | Are the causal relationships described by the model structure justified appropriately? |  |  |
| **S4** | Are the structural assumptions transparent and justified? |  |  |
|  | Are the structural assumptions reasonable given  the overall objective, perspective and scope of the model? |  |  |
| **S5** | Is there a clear definition of the options under  evaluation? |  |  |
|  | Have all feasible and practical options been evaluated? |  |  |
|  | Is there justification for the exclusion of feasible options? |  |  |
| **S6** | Is the chosen model type appropriate given the decision problem and specified causal  relationships within the model? |  |  |
| **S7** | Is the time horizon of the model sufficient to reflect all important differences between options? |  |  |
|  | Is the time horizon of the model, the duration of  treatment and the duration of treatment effect  described and justified? |  |  |
| **S8** | Do the disease states (state transition model) or the pathways (decision tree model) reflect the underlying biological process of the disease in  question and the impact of interventions? |  |  |
| **S9** | Is the cycle length defined and justified in terms  of the natural history of disease? |  |  |
| **DATA (D)** | | | |
| **D1** | Are the data identification methods transparent and appropriate given the objectives of the  model? |  |  |
|  | Where choices have been made between data sources, are these justified appropriately? |  |  |
|  | Has particular attention been paid to identifying data for the important parameters in the model? |  |  |
|  | Has the process of selecting key parameters  been justified and systematic methods used to  identify the most appropriate data? |  |  |
|  | Has the quality of the data been assessed appropriately? |  |  |
|  | Where expert opinion has been used, are the  methods described and justified? |  |  |
| **D2** | Is the pre-model data analysis methodology  based on justifiable statistical and epidemiological techniques? |  |  |
| **D2a** | Is the choice of baseline data described and justified? |  |  |
|  | Are transition probabilities calculated appropriately? |  |  |
|  | Has a half cycle correction been applied to both  cost and outcome? |  |  |
|  | If not, has this omission been justified? |  |  |
| **D2b** | If relative treatment effects have been derived  from trial data, have they been synthesised using  appropriate techniques? |  |  |
|  | Have the methods and assumptions used to extrapolate short-term results to final outcomes  been documented and justified? |  |  |
|  | Have alternative extrapolation assumptions been explored through sensitivity analysis? |  |  |
|  | Have assumptions regarding the continuing effect  of treatment once treatment is complete been  documented and justified? |  |  |
|  | Have alternative assumptions regarding the continuing effect of treatment been explored  through sensitivity analysis? |  |  |
| **D2c** | Are the utilities incorporated into the model  appropriate? |  |  |
|  | Is the source for the utility weights referenced? |  |  |
|  | Are the methods of derivation for the utility weights justified? |  |  |
| **D3** | Have all data incorporated into the model been  described and referenced in sufficient detail? |  |  |
|  | Has the use of mutually inconsistent data been  justified (i.e. are assumptions and choices unclear appropriate)? |  |  |
|  | Is the process of data incorporation transparent? |  |  |
|  | If data have been incorporated as distributions,  has the choice of distribution for each parameter  been described and justified? |  |  |
|  | If data have been incorporated as distributions, is  It clear that second order uncertainty is reflected? |  |  |
| **D4** | Have the four principal types of uncertainty been addressed? |  |  |
|  | If not, has the omission of particular forms of  uncertainty been justified? |  |  |
| **D4a** | Have methodological uncertainties been  addressed by running alternative versions of the  model with different methodological assumptions? |  |  |
| **D4b** | Is there evidence that structural uncertainties have been addressed via sensitivity analysis? |  |  |
| **D4c** | Has heterogeneity been dealt with by running model separately for different sub-groups? |  |  |
| **D4d** | Are the methods of assessment of parameter uncertainty appropriate? |  |  |
|  | If data are incorporated as point estimates, the ranges used for sensitivity analysis stated  clearly and justified? |  |  |
| **Consistency (C)** | | | |
| **C1** | Is there evidence that the mathematical logic of the model has been tested thoroughly before  use? |  |  |
| **C2** | Are the conclusions valid given the data presented? |  |  |
|  | Are any counterintuitive results from the model  explained and justified? |  |  |
|  | If the model has been calibrated against independent data, have any differences been  explained and justified? |  |  |
|  | Have the results of the model been compared with those of previous models and any differences in results explained? |  |  |
