# Supplementary material for: What do we know about managing Dupuytren’s disease cost-effectively?
Source: BMC Musculoskelet Disord. 2018 Jan 25;19:34. doi: 10.1186/s12891-018-1949-2 (PMC5785840; doi:10.1186/s12891-018-1949-2)
Supplement: Supplementary file 4 — List of 65 publications identified in the first stage after removing duplicates. (DOCX 23 kb) [file 12891_2018_1949_MOESM4_ESM.docx]

**Additional file 4: List of 65 publications identified in the first stage after removing duplicates**

1. De Salas-Cansado, M., Antoran, M.B.R., Ramirez, E., and Dudley, A. Utilization of health care resources and cost associated to fasciectomy in Dupuytren's disease in Spain. [Spanish]. Farmacia Hospitalaria, 2013. 37(1): 41-49.

2. De Salas-Cansado, M., Cuadros, M., Del Cerro, M., and Arandes, J.M. Budget impact analysis in Spanish patients with Dupuytren's contracture: Fasciectomy vs. collagenase Clostridium histolyticum. Chirurgie de la Main, 2013. 32(2): 68-73.

3. Donga, P., De Koven, M., Kaplan, F.T.D., Tursi, J., and Lee, W.C. Real-world treatment behavior among patients with dupuytren's contracture: A health insurance claims-based analysis. Value in Health, 2014. 17 (3): A54.

4. Donga, P., Dekoven, M., Thomas Kaplan, F., Tursi, J.P., and Lee, W.C. Costs of collagenase clostridium histolyticum and fasciectomy for dupuytren's contracture. American Journal of Pharmacy Benefits, 2015. 7(1): 24-31.

5. Gerber, R.A., Perry, R., Thompson, R., and Bainbridge, C. Dupuytren's contracture: A retrospective database analysis to assess clinical management and costs in England. BMC Musculoskeletal Disorders, 2011. 12 (no pagination)(73).

6. Gonser, P., Lotter, O., Schaller, H.E., and Jaminet, P. Development of length of stay and reimbursement in elective hand surgery after the introduction of DRGs in Germany. [German]. Handchirurgie Mikrochirurgie Plastische Chirurgie, 2012. 44(5): 306-309.

7. MacAulay, D., Ivanova, J., Birnbaum, H., Sorg, R., and Skodny, P. Direct and indirect costs associated with Dupuytren's contracture. Journal of Medical Economics, 2012. 15(4): 664-671.

8. Maguire, B., Makino, K., Tilden, D., Ballmer, A., and Hart, S. Health care resource requirements and costs during the recovery phase of fasciectomy for the treatment of Dupuytren's contracture: Clinician survey. Value in Health, 2014. 17 (7): A773.

9. Maravic, M. and Beaudreuil, J. Impact on costs of switching one-ray aponeurectomy to percutaneous needle aponeurotomy in Dupuytren's disease: A model analysis. Joint Bone Spine, 2015. 82(4): 264-266.

10. Maravic, M. and Landais, P. Dupuytren's disease in France--1831 to 2001--from description to economic burden. Journal of Hand Surgery, British Volume, 2005. 30(5): 484-7.

11. Mehta, S. and Belcher, H.J.C.R. A single-centre cost comparison analysis of collagenase injection versus surgical fasciectomy for Dupuytren's contracture of the hand. Journal of Plastic, Reconstructive and Aesthetic Surgery, 2014. 67(3): 368-372.

12. Povlsen, B., Shields, A.M., and Bhabra, G.S. Resource utilisation associated with single digit Dupuytren's contracture treated with either surgery or injection of collagenase Clostridium histolyticum. Hand surgery : an international journal devoted to hand and upper limb surgery and related research : journal of the Asia-Pacific Federation of Societies for Surgery of the Hand, 2014. 19(2): 205-209.

13. Sanjuan Cervero, R., Franco Ferrando, N., and Poquet Jornet, J. Use of resources and costs associated with the treatment of Dupuytren's contracture at an orthopedics and traumatology surgery department in Denia (Spain): Collagenase clostridium hystolyticum versus subtotal fasciectomy. BMC Musculoskeletal Disorders, 2013. 14 (no pagination)(293).

14. Skoupa, J. and Hajek, P. Cost of Dupuytren contracture in the Czech Republic. Value in Health, 2011. 14 (7): A307.

15. Atroshi, I., Strandberg, E., Lauritzson, A., Ahlgren, E., and Walden, M. Costs for collagenase injections compared with fasciectomy in the treatment of Dupuytren's contracture: A retrospective cohort study. BMJ Open, 2014. 4 (1) (no pagination)(e004166).

16. Baltzer, H. and Binhammer, P.A. Cost-effectiveness in the management of Dupuytren's contracture: A Canadian cost-utility analysis of current and future management strategies. Bone and Joint Journal, 2013. 95 B(8): 1094-1100.

17. Brazzelli, M., Cruickshank, M., Tassie, E., McNamee, P., Robertson, C., Elders, A., Fraser, C., Hernandez, R., Lawrie, D., and Ramsay, C. Collagenase clostridium histolyticum for the treatment of Dupuytren's contracture: Systematic review and economic evaluation. Health Technology Assessment, 2015. 19(90): 1-201.

18. Chen, N.C., Shauver, M.J., and Chung, K.C. Cost-effectiveness of open partial fasciectomy, needle aponeurotomy, and collagenase injection for Dupuytren contracture. Journal of Hand Surgery, 2011. 36(11): 1826-1834.e32.

19. Eckerdal, D., Nivestam, A., and Dahlin, L.B. Surgical treatment of Dupuytren's disease - Outcome and health economy in relation to smoking and diabetes. BMC Musculoskeletal Disorders, 2014. 15 (1) (no pagination)(117).

20. Ines, M., Silverio, N.M., and Erdogan-Ciftci, E. Cost-minimization analysis of collagenase clostridium histolyticum compared with fasciectomy in patients with Dupuytren's contracture in Portugal. Value in Health, 2011. 14 (7): A310.

21. Lofaro, A. and Adami, M.Z. Cost evaluation of collagenase clostridium histolyticum versus surgery for Dupuytren's contracture. Journal of EuroMed Pharmacy, 2015(5): 20-23.

22. Malone, D.C. and Armstrong, E.P. Cost-effectiveness of collagenase clostridium histolyticum, limited fasciectomy, and percutaneous needle fasciotomy in the treatment of Dupuytren's contracture. Value in Health, 2012. 15 (4): A38-A39.

23. Morhart, M. Pearls and pitfalls of needle aponeurotomy in Dupuytren's disease. Plastic and reconstructive surgery, 2015. 135(3): 817-825.

24. Sanjuan Cervero, R., Carrera-Hueso, F.J., Vazquez-Ferreiro, P., Gomez-Herrero, D., Poquet Jornet, J., Franco Ferrando, N., and Ramon-Barrios, M.A. Clinical Validation of Pharmacoeconomic Models in Dupuytren’s Contracture. Quality in Primary Care, 2015. 23(4): 297-304.

25. Sau, C., Bounthavong, M., Tran, J.N., and Wilson, R.L. Cost-utility analysis of collagenase clostridium histolyticum,limited fasciectomy, and percutaneous needle fasciotomy in Dupuytren's contracture. Value in Health, 2011. 14 (3): A128.

26. Syed, A.M., McFarlane, J., Chester, T., Powers, D., Sibly, F., and Talbot-Smith, A. Clinical efficacy and cost-effectiveness of Clostridium histolyticum collagenase injections in a subpopulation of Dupuytren's contracture patients. European Orthopaedics and Traumatology, 2014. 5(3): 311-316.

27. Wagner, M., Lavoie, L., Hensen, M., Postema, R., and Welner, S. Economic evaluation of collagenase Clostridium histolyticum injection for the treatment of Dupuytren's contracture in Canada. Journal of Population Therapeutics and Clinical Pharmacology, 2014. 21 (1): e126.

28. Webb, J.A. and Stothard, J. Cost minimisation using clinic-based treatment for common hand conditions--a prospective economic analysis. Annals of the Royal College of Surgeons of England, 2009. 91(2): 135-139.

29. Amadio, P.C. What's new in hand surgery. Journal of Bone and Joint Surgery - Series A, 2002. 84(2): 326-330.

30. Amadio, P.C. What's new in hand surgery. Journal of Bone & Joint Surgery - American Volume, 2013. 95(6): 570-4.

31. Anonymous. Xiapex for Dupuytren's contracture. Drug & Therapeutics Bulletin, 2011. 49(12): 138-41.

32. Anonymous. Healthcare Cost, Quality, and Policy: Driving Stakeholder Innovation in Process and Practice. Journal of Population Therapeutics and Clinical Pharmacology, 2014. 21 (1): e116.

33. Bazzocchi, M., Doratiotto, S., Marzio, A., and De Candia, A. Ultrasonographic study of Peyronie's disease. [Italian]. Archivio italiano di urologia, andrologia : organo ufficiale [di] Societa italiana di ecografia urologica e nefrologica / Associazione ricerche in urologia, 2000. 72(4): 376-383.

34. Bebbington, E. and Furniss, D. Linear regression analysis of Hospital Episode Statistics predicts a large increase in demand for elective hand surgery in England. Journal of Plastic, Reconstructive and Aesthetic Surgery, 2015. 68(2): 243-251.

35. Ben-Menachem, E. Data from regulatory studies: What do they tell? What don't they tell? Acta Neurologica Scandinavica, 2005. 112(SUPPL. 181): 21-25.

36. Clayson, D., Verjee-Lorenz, A., Two, R., Gerber, R., and Beaudreuil, J. Translation and linguistic validation methodological implications when the source measure is not English. Value in Health, 2011. 14 (3): A154.

37. Dahlin, L.B., Bainbridge, C., Leclercq, C., Gerber, R.A., Guerin, D., Cappelleri, J.C., Szczypa, P.P., and Dias, J. Dupuytren's disease presentation, referral pathways and resource utilisation in Europe: Regional analysis of a surgeon survey and patient chart review. International Journal of Clinical Practice, 2013. 67(3): 261-270.

38. Das Gupta, K., Lahoda, L.U., Boorboor, P., and Vogt, P.M. Outpatient hand surgery--possibilities and limitations. [German]. Der Chirurg; Zeitschrift fur alle Gebiete der operativen Medizen, 2004. 75(3): 257-264.

39. Descatha, A., Bodin, J., Ha, C., Goubault, P., Lebreton, M., Chastang, J.F., Imbernon, E., Leclerc, A., Goldberg, M., and Roquelaure, Y. Heavy manual work, vibration exposure and Dupuytren's disease? Results from a surveillance program of musculoskeletal disorders. Occupational and Environmental Medicine, 2011. 68: A63.

40. Ding, Y. and Hay, J.W. Economic burden associated with patients diagnosed with Peyronie's disease in the united states. Value in Health, 2014. 17 (3): A157-A158.

41. Donaldson, O.W., Pearson, D., Reynolds, R., and Bhatia, R.K. The association between intraoperative correction of Dupuytren's disease and residual postoperative contracture. The Journal of hand surgery, European volume, 2010. 35(3): 220-223.

42. Ivaldi, L., Perino, M., Gambetta, G., Ferro, A., Colombini, M., Gennaro, M., Mura, G., Carrozza, V., Boetti, M., Baracco, E., and Revetria, P. Day surgery: Five years of experience and activity. [Italian]. Minerva Chirurgica, 2003. 58(2): 149-155.

43. Keilani, M.Y., Paternostro-Sluga, T., Crevenna, R., Zauner-Dungl, A., and Fialka-Moser, V. A report concerning occupational splint supply in an Austrian medical center. [German]. Wiener Medizinische Wochenschrift, 2003. 153(9-10): 222-224.

44. Kuhn, M.A., Payne, W.G., Kierney, P.C., Pu, L.L., Smith, P.D., Siegler, K., Ko, F., Wang, X., and Robson, M.C. Cytokine manipulation of explanted Dupuytren's affected human palmar fascia. International journal of surgical investigation, 2001. 2(6): 443-456.

45. Kurschner, N. and Schiffner-Rohe, J. Challenges in the data collection regarding patient population and treatment costs for non-drug interventions using the example of morbus dupuytren. Value in Health, 2012. 15 (7): A313.

46. Kwan, P. and Brodie, M.J. Phenobarbital for the treatment of epilepsy in the 21st century: A critical review. Epilepsia, 2004. 45(9): 1141-1149.

47. Lalonde, D.H. Reconstruction of the Hand with Wide Awake Surgery. Clinics in Plastic Surgery, 2011. 38(4): 761-769.

48. Lanting, R., Nooraee, N., Werker, P.M., and van den Heuvel, E.R. Patterns of Dupuytren disease in fingers: studying correlations with a multivariate ordinal logit model. Plastic and reconstructive surgery, 2014. 134(3): 483-490.

49. Lanting, R., van den Heuvel, E.R., Westerink, B., and Werker, P.M. Prevalence of Dupuytren disease in The Netherlands. Plastic and reconstructive surgery, 2013. 132(2): 394-403.

50. Maravic, M., Lasbleiz, S., Roulot, E., and Beaudreuil, J. Hospitalization for Dupuytren's disease: A French national descriptive analysis, 2002 to 2009. Orthopaedics and Traumatology: Surgery and Research, 2014. 100(6): 589-592.

51. Peimer, C.A., Pess, G., Skodny, P., Tursi, J., Szczypa, P.P., and Gerber, R.A. Use of collagenase clostridium histolyticum in early-versus advanced-stage Dupuytren's disease results in better outcomes. Annals of the Rheumatic Disease. Conference: Annual European Congress of Rheumatology of the European League Against Rheumatism, EULAR, 2012. 71(no pagination).

52. Seegenschmiedt, M.H., Katalinic, A., Makoski, H.B., Haase, W., Gademann, G., and Hassenstein, E. Radiation therapy of benign diseases: Patterns of care study in Germany. [German]. Strahlentherapie und Onkologie, 1999. 175(11): 541-547.

53. Seegenschmiedt, M.H., Katalinic, A., Makoski, H.B., Haase, W., Gademann, G., and Hassenstein, E. Radiation therapy for benign diseases: Patterns of care study in Germany. International Journal of Radiation Oncology Biology Physics, 2000. 47(1): 195-202.

54. Stein, M.A. and Kanner, A.M. Management of newly diagnosed epilepsy: A practical guide to monotherapy. Drugs, 2009. 69(2): 199-222.

55. Strzelczyk, A., Vogt, H., Hamer, H., and Kramer, G. Recurrent plantar fibromatosis caused by ongoing phenobarbital treatment. Epilepsia, 2009. 50: 205.

56. Uchida, M. Contributions by Guillaume Dupuytren and the treatment of Dupuytren contracture: One aspect of the realization and development of clinical medicine. [Japanese]. Tokyo Jikeikai Medical Journal, 2005. 120(2): 59-72.

57. Voineskos, S.H., Coroneos, C.J., Thoma, A., and Bhandari, M. Measuring and understanding treatment effectiveness in hand surgery. Hand Clinics, 2014. 30(3): 285-292.

58. Wahab, A. Difficulties in treatment and management of epilepsy and challenges in new drug development. Pharmaceuticals, 2010. 3(7): 2090-2110.

59. Waljee, J.F. and Curtin, C. Quality assessment in hand surgery. Hand Clinics, 2014. 30(3): 329-334.

60. Werker, P.M.N., Pess, G.M., Van Rijssen, A.L., and Denkler, K. Correction of contracture and recurrence rates of Dupuytren contracture following invasive treatment: The importance of clear definitions. Journal of Hand Surgery, 2012. 37(10): 2095-2105.

61. Mehta, S. and Belcher, H.J.C.R. Erratum: A single-centre cost comparison analysis of collagenase injection versus surgical fasciectomy for Dupuytren's contracture of the hand (Journal of Plastic, Reconstructive and Aesthetic Surgery (2014) 67 (368-372)). Journal of Plastic, Reconstructive and Aesthetic Surgery, 2014. 67(11): 1612.

62. Wiwanitkit, V. Cost of open partial fasciectomy, needle aponeurotomy, and collagenase injection for Dupuytren contracture. Journal of Hand Surgery, 2012. 37(2): 394.

63. Gu, N.Y., Botteman, M., Gerber, R., Ji, X., Postema, R., Wan, W., Sianos, G., Anthony, I., Cappelleri, J.C., Szczypa, P., and Van Hout, B. Using Discrete Choice Experiments (DCE) to estimate preferencebased utilities for Dupuytren's Contracture (DC). Value in Health, 2012. 15 (4): A50.

64. Gu, N.Y., Botteman, M.F., Gerber, R.A., Ji, X., Postema, R., Wan, Y., Sianos, G., Anthony, I., Cappelleri, J.C., Szczypa, P., and van Hout, B. Eliciting health state utilities for Dupuytren's contracture using a discrete choice experiment. Acta Orthopaedica, 2013. 84(6): 571-8.

65. Li, Y.K., Alolabi, N., Kaur, M.N., and Thoma, A. A systematic review of utilities in hand surgery literature. Journal of Hand Surgery, 2015. 40(5): 997-1005.
