# Supplementary material for: What do we know about managing Dupuytren’s disease cost-effectively?
Source: BMC Musculoskelet Disord. 2018 Jan 25;19:34. doi: 10.1186/s12891-018-1949-2 (PMC5785840; doi:10.1186/s12891-018-1949-2)
Supplement: Supplementary file 5 — Cheers Assessments of included studies. (DOCX 36 kb) [file 12891_2018_1949_MOESM5_ESM.docx]

**Additional file 5- Cheers Assessments of included studies**

| **Study: Chen *et al*., J Hand Surg 2011; 36A: 1826-1834.** | | | |  |
| --- | --- | --- | --- | --- |
| Item No. | Category / item | Reported on page No |  | Comment |
|  | **Title and abstract** |  |  |  |
| 1 | Title | p1826 | Cost-Effectiveness of Open Partial Fasciectomy, Needle Aponeurotomy, and Collagenase Injection for Dupuytren Contracture |  |
| 2 | Abstract | p1826 | **Purpose** We undertook a cost-utility analysis to compare traditional fasciectomy for Dupuytren with 2 new treatments, needle aponeurotomy and collagenase injection.  **Methods** We constructed an expected-value decision analysis model with an arm representing each treatment. A survey was administered to a cohort of 50 consecutive subjects to determine utilities of different interventions. We conducted multiple sensitivity analyses to assess the impact of varying the rate of disease recurrence in each arm of the analysis as well as the cost of the collagenase injection. The threshold for a cost-effective treatment is based on the traditional willingness-to-pay of $50,000 per quality-adjusted life years (QALY) gained.  **Results** The cost of open partial fasciectomy was $820,114 per QALY gained over no treatment. The cost of needle aponeurotomy was $96,474 per QALY gained versus no treatment. When we performed a sensitivity analysis and set the success rate at 100%, the cost of needle aponeurotomy was $49,631. When needle aponeurotomy was performed without surgical center or anesthesia costs and with reduced hand therapy, the cost was $36,570. When a complete collagenase injection series was priced at $250, the cost was $31,856 per QALY gained. When the injection  series was priced at $945, the cost was $49,995 per QALY gained. At the market price of $5,400 per injection, the cost was $166,268 per QALY gained.  **Conclusions** In the current model, open partial fasciectomy is not cost-effective. Needle aponeurotomy is cost-effective if the success rate is high. Collagenase injection is cost effective when priced under $945. |  |
|  | **Introduction** |  |  |  |
| 3 | Background and objectives | p1827 | To undertake a CUA to compare traditional fasciectomy for DD with 2 new treatments, needle aponeurotomy and collagenase injection |  |
|  | **Methods** |  |  |  |
| 4 | Target population and subgroups | p1827 | Adults (mean age: 63 years) with advanced contracture affecting the small and ring fingers |  |
| 5 | Setting and location | p1827 | United States | Setting not clearly described. |
| 6 | Study perspective | p1827 | Societal | No reference to US guidelines/ recommendations regarding perspective. In the discussion, the authors only health care costs are included. |
| 7 | Comparators | p1827 | 1) Collagenease  2) Needle aponeurotomy  3) Open partial fasciectomy |  |
| 8 | Time horizon | p1827 | 20 years |  |
| 9 | Discount rate |  | Not reported | Discounting not reported. Not relevant for the short time horizon. This could have been mentioned for completeness. |
| 10 | Choice of health outcomes | p1828 | QALYs (cost-utility analysis) |  |
| 11 | Measurement of effectiveness |  |  |  |
| 11a | - Single study-based estimates |  | n/a |  |
| 11b | - Synthesis-based estimates | p1828 & Appendix 1 | No systematic review was conducted. Effectiveness estimates were based on survey-based scenario analysis. |  |
| 12 | Measurement and valuation of preference based outcomes | p1828-29 | The utilities were measured by standard gamble technique based on 50 people of the general public (age 50-80 years old) |  |
| 13 | Estimating resources and costs |  |  |  |
| 13a | - Single-study based economic evaluation |  | n/a |  |
| 13b | - Model-based economic evaluation | p1828-29 | *Resource use and costs.* facility, anaesthesia, therapy visits, splint cost, drugs ;  Source: Medicare |  |
| 14 | Currency, price date, and conversion | p1828 | Costs were reported in 2009 US Dollars. |  |
|  | **Economic model** |  |  |  |
| 15 | Choice of model | p1827 & Appendix 1 | Expected value DA model. The model structure is shown in a figure and being described |  |
| 16 | Assumptions | p1827 | Model assumptions were :  I) most subjects of the model are men;  ii) the revision treatment of choice is partial fasciectomy |  |
| 17 | Analytical methods | p1829-30 | Uncertainty was addressed in one-way sensitivity analysis. |  |
|  | **Results** |  |  |  |
| 18 | Study parameters | p1829 | Values, ranges and references for parameters were reported in text and tables (Table 3 and 4). |  |
| 19 | Incremental costs and outcomes |  | Not reported |  |
|  | Characterizing uncertainty |  |  |  |
| 20a | - Single-study based economic evaluation |  | n/a |  |
| 20b | - Model-based economic evaluation | p1829-30 |  | No presentation of sensitivity analyses. One-way only; no multivariate or probabilistic. No graphs/diagrams. |
| 21 | Characterizing heterogeneity |  |  | Heterogeneity was not discussed. |
|  | **Discussion** |  |  |  |
| 22 | Study findings etc | p1829-30 | Each intervention is compared with no treatment, Cost per QALY for each is: Collagenase: $166,268 Fasciectomy: $820,114 Needle fasciotomy: $96,474  Study limitations are:   - Patient –incurred cost not included - Treatment cost after recurrence and complications not included - QALY values have not been adjusted for the probability of further treatment following recurrence/failure - Model structure and pathways not reported - No model robustness was not reported (ie PSA) |  |
|  | **Other** |  |  |  |
| 23 | Source of funding |  | Not mentioned |  |
| 24 | Conflicts of interest |  | Not mentioned |  |

| **Study: Baltzer and Binhammer. The Bone & Joint Journal 2013: 95-B : 1094-1100.** | | | |  |
| --- | --- | --- | --- | --- |
| Item No. | Category / item | Reported on page No |  | Comment |
|  | **Title and abstract** |  |  |  |
| 1 | Title | p1094 | Cost-effectiveness in the management of Dupuytren's contracture: A Canadian cost-utility analysis of current and future management strategies |  |
| 2 | Abstract | p1094 | In Canada, Dupuytren's contracture is managed with partial fasciectomy or percutaneous needle aponeurotomy (PNA). Injectable collagenase will soon be available. The optimal  management of Dupuytren’s contracture is controversial and trade-offs exist between the different methods. Using a cost-utility analysis approach, our aim was to identify the most  cost-effective form of treatment for managing Dupuytren’s contracture it and the threshold at which collagenase is cost-effective. We developed an expected-value decision analysis model for Dupuytren’s contracture affecting a single finger, comparing the cost-effectiveness of fasciectomy, aponeurotomy and collagenase from a societal perspective. Cost effectiveness,  one-way sensitivity and variability analyses were performed using standard thresholds for cost effective treatment ($50 000 to $100 000/QALY gained). Percutaneous needle aponeurotomy was the preferred strategy for managing contractures affecting a single finger. The cost-effectiveness of primary aponeurotomy improved when repeated to treat recurrence. Fasciectomy was not cost-effective. Collagenase was cost-effective relative to and preferred over aponeurotomy at $875 and $470 per course of treatment, respectively.  In summary, our model supports the trend towards non-surgical interventions for managing Dupuytren’s contracture affecting a single finger. Injectable collagenase will only be feasible in our publicly funded healthcare system if it costs significantly less than current United States pricing.. |  |
|  | **Introduction** |  |  |  |
| 3 | Background and objectives | p1094 | To identify the most cost-effective form of treatment for managing DC using a cost-utility analysis |  |
|  | **Methods** |  |  |  |
| 4 | Target population and subgroups | p1095 | Adult men ( mean age : 63 years) with advanced contracture affecting a single finger |  |
| 5 | Setting and location | p1095 | Canada | Setting not clearly described. |
| 6 | Study perspective | p1095 | Healthcare and patient-incurred costs | No reference to Canada guidelines/ recommendations regarding perspective. |
| 7 | Comparators | p1095 | 1) collagenease  2) Percutaneous needle aponeurotomy  3)fasciectomy |  |
| 8 | Time horizon | p1095 | 15 years |  |
| 9 | Discount rate | p1098 | 5% (both cost & utilities) |  |
| 10 | Choice of health outcomes | p1095 | QALY (cost-utility analysis) |  |
| 11 | Measurement of effectiveness |  |  |  |
| 11a | - Single study-based estimates |  | n/a |  |
| 11b | - Synthesis-based estimates | p1096 | No systematic review, no meta-analysis conducted.  Effectiveness estimates were based on a single study. The model parameters were derived through a review of the literature. |  |
| 12 | Measurement and valuation of preference based outcomes | p1096 | Health states utility values were adapted from Chen et al 2011., Besides reference to this paper, no further description of method/technique used is given. | A brief description of population/technique used in source publications could have been added. |
| 13 | Estimating resources and costs |  |  |  |
| 13a | - Single-study based economic evaluation |  | n/a |  |
| 13b | - Model-based economic evaluation | p1097-8 | *Resource use and costs.* : hospital facility, allied healthcare and hand therapy fees, equipment and pharmaceutical costs, medication, costs related to complications included the cost of salvage PF, repair of damaged digital nerves and additional hand therapy, productivity loss, parking cost for appointments, income loss;  Source: Ontario Health Insurance Program |  |
| 14 | Currency, price date, and conversion | p1095 | Costs were reported in 2011 US Dollars. |  |
|  | **Economic model** |  |  |  |
| 15 | Choice of model | p1095 | Expected value decision analytic model model. Structure of the model shown in Figure 1. |  |
| 16 | Assumptions | p1096 | Model assumptions were :  • cost of collagenase represented an entire course of treatment • wound healing problems, infection and haematoma following PF or PNA are relatively short-lived |  |
| 17 | Analytical methods | p1096 | Uncertainty was addressed with a mutlivariate sensitivity on patterns of clinical practice and one-way sensitivity analysis on key assumptions and model parameters. | . |
|  | **Results** |  |  |  |
| 18 | Study parameters | p1097 | Baseline probabilities are presented in Table 3. |  |
| 19 | Incremental costs and outcomes | p1098 | QALYs and costs associated with each strategy and ICERs are presented in Tables 5&6. |  |
|  | Characterizing uncertainty |  |  |  |
| 20a | - Single-study based economic evaluation |  | n/a |  |
| 20b | - Model-based economic evaluation | p1098 | Results of one-way sensitivity analyses were presented, Figure 3.  •Model not sensitive to patient-incurred costs  • Model sensitive to high utility values for all health states |  |
| 21 | Characterizing heterogeneity |  |  | Heterogeneity was not discussed. |
|  | **Discussion** |  |  |  |
| 22 | Study findings etc | p1098-9 | Fasciectomy was dominated. Collagenase cost $284,383 per QALY compared with percutaneous needle aponeurotomy.  Study limitations are:  • This model was analysed for patients receiving treatment on a single finger and therefore the results may not be generalizable to patient with multiple fingers affected  •QALY values have not been adjusted for the probability of further treatment following recurrence/failure |  |
|  | **Other** |  |  |  |
| 23 | Source of funding |  | Not mentioned |  |
| 24 | Conflicts of interest |  | Not mentioned |  |

| **Study: Brazzelli M *et al.* Health Technology Assessment 2015: 19 (90)** | | | |  |
| --- | --- | --- | --- | --- |
| Item No. | Category / item | Reported on page No |  | Comment |
|  | **Title and abstract** |  |  |  |
| 1 | Title | v | Collagenase clostridium histolyticum for the treatment of Dupuytren’s contracture: systematic review and economic evaluation |  |
| 2 | Abstract | v | Background: Dupuytren’s disease is a slowly progressive condition of the hand, characterised by the formation of nodules in the palm that gradually develop into fibrotic cords. Contracture of the cords produces deformities of the fingers. Surgery is recommended for moderate and severe contractures, but complications and/or recurrences are frequent. Collagenase clostridium histolyticum (CCH) has been developed as a minimally invasive alternative to surgery for some patients.  Objectives: To assess the clinical effectiveness and cost-effectiveness of collagenase as an alternative to surgery for adults with Dupuytren’s contracture with a palpable cord.  Data sources: We searched all major electronic databases from 1990 to February 2014.  Review methods: Randomised controlled trials (RCTs), non-randomised comparative studies and observational studies involving collagenase and/or surgical interventions were considered. Two reviewers ndependently extracted data and assessed risk of bias of included studies. A de novo Markov model was developed to assess cost-effectiveness of collagenase, percutaneous needle fasciotomy (PNF) and limited fasciectomy (LF). Results were reported as incremental cost per quality-adjusted life-year (QALY) gained. Deterministic and probabilistic sensitivity analyses were undertaken to investigate model and parameter uncertainty.  Results: Five RCTs comparing collagenase with placebo (493 participants), three RCTs comparing surgical techniques (334 participants), two non-randomised studies comparing collagenase and surgery (105 participants), five non-randomised comparative studies assessing various surgical procedures (3571 participants) and 15 collagenase case series (3154 participants) were included. Meta-analyses of RCTs assessing CCH versus placebo were performed. Joints randomised to collagenase were more likely to achieve clinical success. Collagenase-treated participants experienced significant reduction in contracture and an increased range of motion compared with placebo-treated participants. Participants treated with collagenase also experienced significantly more adverse events, most of which were mild or moderate. Four serious adverse events were observed in the collagenase group: two tendon ruptures, one pulley rupture and one complex regional pain syndrome. Two tendon ruptures were also reported in two collagenase case series. Non-randomised studies comparing collagenase with surgery produced variable results and were at high risk of bias. Serious adverse events across surgery studies were low. Recurrence rates ranged from 0% (at 90 days) to 100% (at 8 years) for collagenase and from 0% (at 2.7 years for fasciectomy) to 85% (at 5 years for PNF) for surgery. The results of the de novo economic analysis show that PNF was the cheapest treatment option, whereas LF generated the greatest QALY gains. Collagenase was more costly and generated fewer QALYs compared with LF. LF was £1199 more costly and generated an additional 0.11 QALYs in comparison with PNF. The incremental cost-effectiveness ratio was £10,871 per QALY gained. Two subgroup analyses were conducted for a population of patients with moderate and severe disease and up to two joints affected. In both subgroup analyses, collagenase remained dominated.  Limitations: The main limitation of the review was the lack of head-to-head RCTs comparing collagenase with surgery and the limited evidence base for estimating the effects of specific surgical procedures (fasciectomy and PNF). Substantial differences across studies further limited the comparability of available evidence. The economic model was derived from a naive indirect comparison and was hindered by a lack of suitable data. In addition, there was considerable uncertainty about the appropriateness of many assumptions and parameters used in the model.  Conclusions: Collagenase was significantly better than placebo. There was no evidence that collagenase was clinically better or worse than surgical treatments. LF was the most cost-effective choice to treat moderate to severe contractures, whereas collagenase was not. However, the results of the cost–utility analysis are based on a naive indirect comparison of clinical effectiveness, and a RCT is required to confirm or refute these findings. |  |
|  | **Introduction** |  |  |  |
| 3 | Background and objectives | p47 | To assess the clinical effectiveness and cost-effectiveness of collagenase as an alternative to surgery for adults with DC with a palpable cord. |  |
|  | **Methods** |  |  |  |
| 4 | Target population and subgroups | p57 | Adults (mean age, 63 years ) with moderate to severe disease |  |
| 5 | Setting and location | p57 | United Kingdom |  |
| 6 | Study perspective | p50 | NHS and personal social services | Reference to UK guidelines/ recommendations regarding perspective. |
| 7 | Comparators | p58 | 1) collagenease  2) Percutaneous needle fasciotomy  3) Limited fasciectomy |  |
| 8 | Time horizon | p70 | 37 year (lifetime) |  |
| 9 | Discount rate | p69 | 3.5% (both cost & utilities) |  |
| 10 | Choice of health outcomes | p67 | QALY (cost-utility analysis) |  |
| 11 | Measurement of effectiveness |  |  |  |
| 11a | - Single study-based estimates |  | n/a |  |
| 11b | - Synthesis-based estimates | p66 | Effectiveness estimates were based on a single study. The model parameters were derived through a review of the literature. |  |
| 12 | Measurement and valuation of preference based outcomes | p66 | Utilities valuation was based on the general population (1,745 UK respondents) using a discrete choice experiment based on Gu et al., 2013. | A description of population/technique used in source is provided. |
| 13 | Estimating resources and costs |  |  |  |
| 13a | - Single-study based economic evaluation |  | n/a |  |
| 13b | - Model-based economic evaluation | p65 | *Resource use and costs.* : Injection cost, administration visit, finger extension examination, splint, physiotherapy appointment; Source: UK NHS reference cost |  |
| 14 | Currency, price date, and conversion | p61 | Costs were reported in 2012/13 UK Pounds |  |
|  | **Economic model** |  |  |  |
| 15 | Choice of model | p58 &63 | Markov model. Structure of the model shown in Figures 30 & 31. |  |
| 16 | Assumptions | p69 | Model assumptions were :  (a) The modelled patient cohort had three joints affected by DC. (b) 1.6 CCH injections were required to treat each joint in separate visits to hospital outpatient clinics. (c) Equivalent recurrence rates were applied to first-, second- and third-line treatment. (d) Equivalent treatment success and treatment failure rates were applied to first-, second- and third-line treatment. (e) Patients who failed after LF did not proceed for any further treatment. (f) The proportion of patients proceeding for further treatment in the CCH strategy was assumed to be the same as LF. (g) All patients received LF for third-line treatment. |  |
| 17 | Analytical methods | p70-71 | Uncertainty was addressed with a deterministic sensitivity analysis on various parameters; Probabilistic sensitivity analysis was also performed on various parameters;  cycle length: 6 months (half-cycle corrections) | . |
|  | **Results** |  |  |  |
| 18 | Study parameters | p59-62 | Study parameters are reported in Tables 14-17 parameter distribution: gamma |  |
| 19 | Incremental costs and outcomes | p71 | QALYs and costs associated with each strategy and ICERs are presented in Tables 23. |  |
|  | Characterizing uncertainty |  |  |  |
| 20a | - Single-study based economic evaluation |  | n/a |  |
| 20b | - Model-based economic evaluation | p73-76 | Results of sensitivity analyses were presented, Table 24.  •cost-effectiveness results are primarily driven by treatment effectiveness • model is sensitive to incremental cost of 1st line treatment |  |
| 21 | Characterizing heterogeneity | p72 | In patients with moderate and severe disease, PNF is the preferred option |  |
|  | **Discussion** |  |  |  |
| 22 | Study findings etc | p82 | Collagenase was dominated. Fasciectomy cost £10,871 per QALY gained in comparison with percutaneous fasciotomy.  Study limitations:  •No direct comparisons of the surgical treatments have been performed using a RCT study design  • no reliable indirect comparisons can be made owing to the lack of a common comparator  • differences in the patient characteristics  • economic model built from a naïve indirect comparison and as such represents a departure from the NICE reference case |  |
|  | **Other** |  |  |  |
| 23 | Source of funding | vi | The National Institute for Health Research Health Technology Assessment Programme |  |
| 24 | Conflicts of interest |  | Mentioned |  |

| **Study: Sau *et al*. Value in Health 2011: 14 (3); A128.** | | | |  |
| --- | --- | --- | --- | --- |
| Item No. | Category / item | Reported on page No |  | Comment |
|  | **Title and abstract** |  |  |  |
| 1 | Title | A128 | Cost-utility analysis of Collagenase Clostridium Histolyticum, Limited Fasciectomy, and Percutaneous Needle Fasciotomy in Dupuytren's Contracture |  |
| 2 | Abstract | A128 | **OBJECTIVES**: To assess the cost-effectiveness of limited fasciectomy (LF), percutaneous needle fasciotomy (PNF), and collagenase clostridium histolyticum (CCH) for the treatment of Dupuytren’s contracture. **METHODS**: A Markov model was developed developed to simulate Dupuytren’s contracture progression and estimate clinical/economic implications of LF, PNF, and CCH treatments from a US healthcare payer perspective. Transition probabilities were assumed to follow a beta distribution and were estimated based on results from randomized, clinical trials. Health state utilities and direct costs of therapies were assumed to follow a gamma distribution and obtained from published sources. Half-cycle correction was used with a 1-year cycle length over a 10-year time horizon. One-way sensitivity analyses were performed on relevant variables to test the robustness of the model. Probabilistic sensitivity analysis was performed using 10,000 trial simulations for all variables and results were presented as acceptability curves. The model used a discount rate of 3% per annum and reported in 2010 $US dollars. Primary outcomes evaluated incremental cost-effectiveness ratios. **RESULTS**: Of the 3 treatment decisions, LF was the dominant strategy. PNF and CCH were estimated to cost an additional $247 and $1844 compared to LF, respectively. An expected difference of -0.1 and -0.04 quality-adjusted life years (QALYs) were projected for PNF and CCH relative to LF, respectively. In the one-way sensitivity analysis, the model was sensitive to direct cost of LF with a break-even point of $2000 compared to PNF. The acceptability curve showed that LF had a higher probability of being cost-effective compared to other treatment modalities across a WTP threshold of $0 to $500,000. **CONCLUSIONS**: Across a WTP threshold between $0 and $500,000, LF was the most cost-effective therapy for the treatment of Dupuytren’s contracture compared to PNF and CCH. However, the cost of surgery was sensitive in our model which may vary from site to site. |  |
|  | **Introduction** |  |  |  |
| 3 | Background and objectives | A128 | To assess the cost-effectiveness of LF, PNF and CCH for the treatment of DC |  |
|  | **Methods** |  |  |  |
| 4 | Target population and subgroups | poster | Adults( mean age :50 years) with advanced contracture |  |
| 5 | Setting and location | poster | United States, | Setting not clearly described. |
| 6 | Study perspective | poster | Healthcare Payer | No reference to US guidelines/ recommendations regarding perspective. |
| 7 | Comparators | poster | 1) collagenase,  2) limited fasciectomy  3) Percutaneous needle fasciotomy |  |
| 8 | Time horizon | poster | 10 years |  |
| 9 | Discount rate | poster | 3% (both cost & utilities) |  |
| 10 | Choice of health outcomes | poster | QALY (cost-utility analysis) |  |
| 11 | Measurement of effectiveness |  |  |  |
| 11a | - Single study-based estimates |  | n/a |  |
| 11b | - Synthesis-based estimates | poster | No systematic review, no meta-analysis conducted.  Effectiveness estimates were based on a single study. The model parameters were derived through a review of the literature. Cost & Utility distribution: gamma |  |
| 12 | Measurement and valuation of preference based outcomes | poster | Utilities valuation was based on patients with carpal tunnel syndrome from Atroshi et al., 2007. Besides reference to this paper, no further description of method/technique used is given. | A brief description of population/technique used in source publications could have been added. |
| 13 | Estimating resources and costs |  |  |  |
| 13a | - Single-study based economic evaluation |  | n/a |  |
| 13b | - Model-based economic evaluation | poster | *Resource use and costs.* : medication, physician visits, surgical procedures, hand therapy, adverse events;  Source: Medicare |  |
| 14 | Currency, price date, and conversion | poster | Costs were reported in 2010 US Dollars. |  |
|  | **Economic model** |  |  |  |
| 15 | Choice of model | poster | Markov model. Structure of the model shown in Figure 1. |  |
| 16 | Assumptions | poster | Model assumptions were :   - Patients surgically treated with LF will undergo a full course of hand therapy - Non-serious adverse events unlikely to impact outcome measures - Patients with DC have similar health utility scores as patients with carpal tunnel syndrome - One-year cycle length reflective of assumption that clinicians would wait at least one year to assess results of an intervention before initiating another |  |
| 17 | Analytical methods | poster | Uncertainty was addressed in one-way sensitivity analysis.  Cycle length: 1 year (half-cycle corrections) | . |
|  | **Results** |  |  |  |
| 18 | Study parameters | poster | Model transition probabilities are presented in Table 1. |  |
| 19 | Incremental costs and outcomes | poster | QALYs and costs associated with each strategy and ICERs are presented in Table 2. |  |
|  | Characterizing uncertainty |  |  |  |
| 20a | - Single-study based economic evaluation |  | n/a |  |
| 20b | - Model-based economic evaluation | poster | Results of one-way sensitivity analyses were presented, Figure 2.  Model sensitive to direct cost of Limited Fasciectomy | No multivariate or probabilistic sensitivity analyses were presented. |
| 21 | Characterizing heterogeneity |  |  | Heterogeneity was not discussed. |
|  | **Discussion** |  |  |  |
| 22 | Study findings etc | poster | •Collagenase and percutaneous needle fasciotomy were dominated by limited fasciectomy  •The model is sensitive to direct cost of LF. Costs of alternative therapies did not have a significant impact on results.  Limitations of the study include:  •Few trials available comparing efficacy data of treatment modalities •Efficacy endpoints defined differently among trials •Health utility scores extrapolated from patients with carpal tunnel syndrome |  |
|  | **Other** |  |  |  |
| 23 | Source of funding |  | Not mentioned |  |
| 24 | Conflicts of interest |  | Not mentioned |  |
