# Supplementary material for: What do we know about managing Dupuytren’s disease cost-effectively?
Source: BMC Musculoskelet Disord. 2018 Jan 25;19:34. doi: 10.1186/s12891-018-1949-2 (PMC5785840; doi:10.1186/s12891-018-1949-2)
Supplement: Supplementary file 6 — Quality assessments framework to the modelling studies [19]. (DOCX 40 kb) [file 12891_2018_1949_MOESM6_ESM.docx]

**Additional file 6- Quality assessments framework to the modelling studies [19]**

| **Study: Chen et al., J Hand Surg 2011; 36A: 1826-1834.** | | | |
| --- | --- | --- | --- |
| **Quality**  **criteria** | **Question(s) for critical appraisal** | **Response** | **Comments** |
| **Structure (S)** | | | |
| **S1** | Is there a clear statement of the decision problem? | YES | To undertake a cost-utility analysis to compare traditional fasciectomy for Dupuytren with 2 new treatments, needle aponeurotomy and collagenase injection |
|  | Is the objective of the evaluation and model specified and consistent with the stated decision  problem? | YES |  |
|  | Is the primary decision maker specified? | NO | None stated |
| **S2** | Is the perspective of the model stated clearly? | YES | It’s the societal perspective |
|  | Are the model inputs consistent with the stated  perspective? | NO | Cost born by patients and out-off pocket expenses are not included. Authors mentioned societal perspective but only costs of Medicare included. |
|  | Has the scope of the model been stated and justified? | YES | Assumptions although stated, have not been explained sufficiently in the context of available evidence |
|  | Are the outcomes of the model consistent with  the perspective, scope and overall objective of  the model? | NO | Only direct treatment costs are considered although the analysis perspective is a societal one |
| **S3** | Has the evidence regarding the model structure been described?  Is the structure of the model consistent with a coherent theory of the health condition under evaluation? | NO |  |
|  | Are the sources of data used to develop the structure of the model specified? | NO |  |
|  | Are the causal relationships described by the model structure justified appropriately? | NO |  |
| **S4** | Are the structural assumptions transparent and justified? | NO |  |
|  | Are the structural assumptions reasonable given  the overall objective, perspective and scope of the model? | NO |  |
| **S5** | Is there a clear definition of the options under  evaluation? | YES |  |
|  | Have all feasible and practical options been evaluated? | NO | Decision analysis has been focused only on 3 treatments |
|  | Is there justification for the exclusion of feasible options? | NO | Not given |
| **S6** | Is the chosen model type appropriate given the decision problem and specified causal  relationships within the model? | NO | This seems to be a decision tree with no time-variant component and hence not appropriate to model a chronic condition. |
| **S7** | Is the time horizon of the model sufficient to reflect all important differences between options? | YES | The model takes a lifetime horizon (20 years on the mean base age of 63 years) |
|  | Is the time horizon of the model, the duration of  treatment and the duration of treatment effect  described and justified? | NO | Duration of treatment and duration of treatment effect have been adequately discussed |
| **S8** | Do the disease states (state transition model) or the pathways (decision tree model) reflect the underlying biological process of the disease in  question and the impact of interventions? | NO | Little detail to assess this. |
| **S9** | Is the cycle length defined and justified in terms  of the natural history of disease? | N/A |  |
| **DATA (D)** | | | |
| **D1** | Are the data identification methods transparent and appropriate given the objectives of the  model? | YES | Systematic review conducted |
|  | Where choices have been made between data sources, are these justified appropriately? | NO | Not clear how effectiveness estimated were estimated |
|  | Has particular attention been paid to identifying data for the important parameters in the model? | YES | Recurrence rates and complication rates have been retrieved from literature evidence |
|  | Has the process of selecting key parameters  been justified and systematic methods used to  identify the most appropriate data? | NO | Not specified |
|  | Has the quality of the data been assessed appropriately? | NO | Not specified |
|  | Where expert opinion has been used, are the  methods described and justified? | N/A |  |
| **D2** | Is the pre-model data analysis methodology  based on justifiable statistical and epidemiological techniques? | NO |  |
| **D2a** | Is the choice of baseline data described and justified? | NO |  |
|  | Are transition probabilities calculated appropriately? | N/A |  |
|  | Has a half cycle correction been applied to both  cost and outcome? | N/A |  |
|  | If not, has this omission been justified? | N/A |  |
| **D2b** | If relative treatment effects have been derived  from trial data, have they been synthesised using  appropriate techniques? | N/A |  |
|  | Have the methods and assumptions used to extrapolate short-term results to final outcomes  been documented and justified? | NO |  |
|  | Have alternative extrapolation assumptions been explored through sensitivity analysis? | NO | No sensitivity analysis has been performed to alternative extrapolation assumptions |
|  | Have assumptions regarding the continuing effect  of treatment once treatment is complete been  documented and justified? | NO | Although the model has taken a lifetime approach, the implementation is not appropriate for this condition. |
|  | Have alternative assumptions regarding the continuing effect of treatment been explored  through sensitivity analysis? | NO |  |
| **D2c** | Are the utilities incorporated into the model  appropriate? | YES | The utilities in the model were based on a survey which included 13 standard gamble scenarios based on the decision tree. Survey members were elected from the general public, aged between 50-80 years old. Utilities ranged from 0.971 to 0.994. |
|  | Is the source for the utility weights referenced? | YES | SG exercise |
|  | Are the methods of derivation for the utility weights justified? | YES |  |
| **D3** | Have all data incorporated into the model been  described and referenced in sufficient detail? | NO |  |
|  | Has the use of mutually inconsistent data been  justified (i.e. are assumptions and choices unclear appropriate)? | NO |  |
|  | Is the process of data incorporation transparent? | NO |  |
|  | If data have been incorporated as distributions,  has the choice of distribution for each parameter  been described and justified? | N/A |  |
|  | If data have been incorporated as distributions, is  It clear that second order uncertainty is reflected? | N/A |  |
| **D4** | Have the four principal types of uncertainty been addressed? | NO | Only parameter uncertainty was performed through univariate sensitivity analysis on recurrence rate, complication rate and various cost items |
|  | If not, has the omission of particular forms of  uncertainty been justified? | NO | Omission of other form of uncertainty has not been explained |
| **D4a** | Have methodological uncertainties been  addressed by running alternative versions of the  model with different methodological assumptions? | NO |  |
| **D4b** | Is there evidence that structural uncertainties have been addressed via sensitivity analysis? | NO |  |
| **D4c** | Has heterogeneity been dealt with by running model separately for different sub-groups? | NO |  |
| **D4d** | Are the methods of assessment of parameter uncertainty appropriate? | NO |  |
|  | If data are incorporated as point estimates, the ranges used for sensitivity analysis stated  clearly and justified? | NO | Range of the value parameters is partly presented but not always justified |
| **Consistency (C)** | | | |
| **C1** | Is there evidence that the mathematical logic of the model has been tested thoroughly before  use? | NO | No consistency checks are reported |
| **C2** | Are the conclusions valid given the data presented? | YES | Model conclusions are explicable |
|  | Are any counterintuitive results from the model  explained and justified? | N/A |  |
|  | If the model has been calibrated against independent data, have any differences been  explained and justified? | N/A |  |
|  | Have the results of the model been compared with those of previous models and any differences in results explained? | NO | No comparison to previous models has been made |
| **Study: Baltzer and Binhammer. The Bone & Joint Journal 2013: 95-B : 1094-1100.** | | | |
| **Quality**  **criteria** | **Question(s) for critical appraisal** | **Response** | **Comments** |
| **Structure (S)** | | | |
| **S1** | Is there a clear statement of the decision problem? | YES | To develop an expected-value decision analysis model for Dupuytren’s contracture affecting a single finger, comparing the cost-effectiveness of fasciectomy, aponeurotomy and collagenase |
|  | Is the objective of the evaluation and model specified and consistent with the stated decision  problem? | YES |  |
|  | Is the primary decision maker specified? | NO |  |
| **S2** | Is the perspective of the model stated clearly? | YES | It’s the societal perspective |
|  | Are the model inputs consistent with the stated  perspective? | YES | Both healthcare cost as well as patient-incurred cost, including lost productivity associated with recovery from surgery were considered |
|  | Has the scope of the model been stated and justified? | YES |  |
|  | Are the outcomes of the model consistent with  the perspective, scope and overall objective of  the model? | YES |  |
| **S3** | Has the evidence regarding the model structure been described?  Is the structure of the model consistent with a coherent theory of the health condition under evaluation? | NO | This is not clearly reported in the manuscript |
|  | Are the sources of data used to develop the structure of the model specified? | YES | Based on previous US study |
|  | Are the causal relationships described by the model structure justified appropriately? | NO |  |
| **S4** | Are the structural assumptions transparent and justified? | NO | Not clear whether the structure is appropriate. |
|  | Are the structural assumptions reasonable given  the overall objective, perspective and scope of the model? | NO |  |
| **S5** | Is there a clear definition of the options under  evaluation? | YES |  |
|  | Have all feasible and practical options been evaluated? | NO | 3 treatments have been considered |
|  | Is there justification for the exclusion of feasible options? | NO |  |
| **S6** | Is the chosen model type appropriate given the decision problem and specified causal  relationships within the model? | NO | Decision analytic model used for chronic condition. Time component not implicitly included. |
| **S7** | Is the time horizon of the model sufficient to reflect all important differences between options? | YES | The model took a lifetime horizon (15 years assuming mean age of 63 years) but not correctly implemented. |
|  | Is the time horizon of the model, the duration of  treatment and the duration of treatment effect  described and justified? | NO | Authors do not justify duration treatment effect |
| **S8** | Do the disease states (state transition model) or the pathways (decision tree model) reflect the underlying biological process of the disease in  question and the impact of interventions? | YES | Pathways of treatment are being discussed |
| **S9** | Is the cycle length defined and justified in terms  of the natural history of disease? | N/A |  |
| **DATA (D)** | | | |
| **D1** | Are the data identification methods transparent and appropriate given the objectives of the  model? | YES |  |
|  | Where choices have been made between data sources, are these justified appropriately? | NO | Evidence synthesis of baseline probabilities not correct methodology |
|  | Has particular attention been paid to identifying data for the important parameters in the model? | YES | Systematic review of baseline probabilities |
|  | Has the process of selecting key parameters  been justified and systematic methods used to  identify the most appropriate data? | NO |  |
|  | Has the quality of the data been assessed appropriately? | NO |  |
|  | Where expert opinion has been used, are the  methods described and justified? | N/A |  |
| **D2** | Is the pre-model data analysis methodology  based on justifiable statistical and epidemiological techniques? | NO |  |
| **D2a** | Is the choice of baseline data described and justified? | YES |  |
|  | Are transition probabilities calculated appropriately? | N/A |  |
|  | Has a half cycle correction been applied to both  cost and outcome? | N/A |  |
|  | If not, has this omission been justified? | N/A |  |
| **D2b** | If relative treatment effects have been derived  from trial data, have they been synthesised using  appropriate techniques? | NO | No details have been provided about the how health state utilities have been adapted from published literature |
|  | Have the methods and assumptions used to extrapolate short-term results to final outcomes  been documented and justified? | NO | Assumption are mentioned but not always justified |
|  | Have alternative extrapolation assumptions been explored through sensitivity analysis? | NO |  |
|  | Have assumptions regarding the continuing effect  of treatment once treatment is complete been  documented and justified? | NO | The model has taken a lifetime approach but not correctly implemented |
|  | Have alternative assumptions regarding the continuing effect of treatment been explored  through sensitivity analysis? | NO |  |
| **D2c** | Are the utilities incorporated into the model  appropriate? | YES | The discussion around the use of utilities applied in the model is not extensive enough so that the reader cannot judge its appropriateness |
|  | Is the source for the utility weights referenced? | YES |  |
|  | Are the methods of derivation for the utility weights justified? | NO |  |
| **D3** | Have all data incorporated into the model been  described and referenced in sufficient detail? | YES |  |
|  | Has the use of mutually inconsistent data been  justified (i.e. are assumptions and choices unclear appropriate)? | N/A |  |
|  | Is the process of data incorporation transparent? | YES |  |
|  | If data have been incorporated as distributions,  has the choice of distribution for each parameter  been described and justified? | N/A |  |
|  | If data have been incorporated as distributions, is  It clear that second order uncertainty is reflected? | N/A |  |
| **D4** | Have the four principal types of uncertainty been addressed? | NO | Only parameter uncertainty was performed through one-way sensitivity analysis |
|  | If not, has the omission of particular forms of  uncertainty been justified? | NO | Omission of other forms of uncertainty has not been explained |
| **D4a** | Have methodological uncertainties been  addressed by running alternative versions of the  model with different methodological assumptions? | NO |  |
| **D4b** | Is there evidence that structural uncertainties have been addressed via sensitivity analysis? | NO |  |
| **D4c** | Has heterogeneity been dealt with by running model separately for different sub-groups? | YES |  |
| **D4d** | Are the methods of assessment of parameter uncertainty appropriate? | NO |  |
|  | If data are incorporated as point estimates, the ranges used for sensitivity analysis stated  clearly and justified? | NO | No explanation about the ranges used provided |
| **Consistency (C)** | | | |
| **C1** | Is there evidence that the mathematical logic of the model has been tested thoroughly before  use? | NO |  |
| **C2** | Are the conclusions valid given the data presented? | YES |  |
|  | Are any counterintuitive results from the model  explained and justified? | N/A |  |
|  | If the model has been calibrated against independent data, have any differences been  explained and justified? | N/A |  |
|  | Have the results of the model been compared with those of previous models and any differences in results explained? | YES |  |

| **Study: Brazzelli M et al. Health Technology Assessment 2015: 19 (90)** | | | |
| --- | --- | --- | --- |
| **Quality**  **criteria** | **Question(s) for critical appraisal** | **Response** | **Comments** |
| **Structure (S)** | | | |
| **S1** | Is there a clear statement of the decision problem? | YES | To assess the clinical effectiveness and cost-effectiveness of collagenase as an alternative to surgery for adults with DC with a palpable cord. |
|  | Is the objective of the evaluation and model specified and consistent with the stated decision  problem? | YES |  |
|  | Is the primary decision maker specified? | NO | None stated |
| **S2** | Is the perspective of the model stated clearly? | YES | It’s the National Health System (NHS) and Personal Social Services (PSS) |
|  | Are the model inputs consistent with the stated  perspective? | YES |  |
|  | Has the scope of the model been stated and justified? | YES |  |
|  | Are the outcomes of the model consistent with  the perspective, scope and overall objective of  the model? | YES |  |
| **S3** | Has the evidence regarding the model structure been described?  Is the structure of the model consistent with a coherent theory of the health condition under evaluation? | YES |  |
|  | Are the sources of data used to develop the structure of the model specified? | YES | All sources of data are clearly specified |
|  | Are the causal relationships described by the model structure justified appropriately? | NO |  |
| **S4** | Are the structural assumptions transparent and justified? | YES |  |
|  | Are the structural assumptions reasonable given  the overall objective, perspective and scope of the model? | YES |  |
| **S5** | Is there a clear definition of the options under  evaluation? | YES |  |
|  | Have all feasible and practical options been evaluated? | NO | The economic analysis focuses on three strategies collagenase, percutaneous needle fasciotomy and limited fasciectomy. Authors provided justification of exclusion of other available treatment options. |
|  | Is there justification for the exclusion of feasible options? | NO |  |
| **S6** | Is the chosen model type appropriate given the decision problem and specified causal  relationships within the model? | YES | A markov model was used, health states are being described and justified as well as the limitation of the model |
| **S7** | Is the time horizon of the model sufficient to reflect all important differences between options? | YES |  |
|  | Is the time horizon of the model, the duration of  treatment and the duration of treatment effect  described and justified? | YES | The model took a lifetime horizon, following the cohort from age 63 to 100 years. |
| **S8** | Do the disease states (state transition model) or the pathways (decision tree model) reflect the underlying biological process of the disease in  question and the impact of interventions? | YES |  |
| **S9** | Is the cycle length defined and justified in terms  of the natural history of disease? | YES | Cycle length is 6 months and included a half-cycle correction |
| **DATA (D)** | | | |
| **D1** | Are the data identification methods transparent and appropriate given the objectives of the  model? | YES |  |
|  | Where choices have been made between data sources, are these justified appropriately? | YES |  |
|  | Has particular attention been paid to identifying data for the important parameters in the model? | YES |  |
|  | Has the process of selecting key parameters  been justified and systematic methods used to  identify the most appropriate data? | YES |  |
|  | Has the quality of the data been assessed appropriately? | NO |  |
|  | Where expert opinion has been used, are the  methods described and justified? | YES |  |
| **D2** | Is the pre-model data analysis methodology  based on justifiable statistical and epidemiological techniques? | NO |  |
| **D2a** | Is the choice of baseline data described and justified? | YES |  |
|  | Are transition probabilities calculated appropriately? | YES |  |
|  | Has a half cycle correction been applied to both  cost and outcome? | YES |  |
|  | If not, has this omission been justified? | N/A |  |
| **D2b** | If relative treatment effects have been derived  from trial data, have they been synthesised using  appropriate techniques? | N/A |  |
|  | Have the methods and assumptions used to extrapolate short-term results to final outcomes  been documented and justified? | YES |  |
|  | Have alternative extrapolation assumptions been explored through sensitivity analysis? | YES |  |
|  | Have assumptions regarding the continuing effect  of treatment once treatment is complete been  documented and justified? | YES |  |
|  | Have alternative assumptions regarding the continuing effect of treatment been explored  through sensitivity analysis? | YES |  |
| **D2c** | Are the utilities incorporated into the model  appropriate? | YES | Health state preferences were elicited via an internet survey from 1745 respondents resident in the UK |
|  | Is the source for the utility weights referenced? | YES |  |
|  | Are the methods of derivation for the utility weights justified? | YES |  |
| **D3** | Have all data incorporated into the model been  described and referenced in sufficient detail? | YES |  |
|  | Has the use of mutually inconsistent data been  justified (i.e. are assumptions and choices unclear appropriate)? | N/A |  |
|  | Is the process of data incorporation transparent? | YES |  |
|  | If data have been incorporated as distributions,  has the choice of distribution for each parameter  been described and justified? | YES | Parameter distributions are presented in detail but have not been justified |
|  | If data have been incorporated as distributions, is  it clear that second order uncertainty is reflected? | YES |  |
| **D4** | Have the four principal types of uncertainty been addressed? | YES |  |
|  | If not, has the omission of particular forms of  uncertainty been justified? | N/A |  |
| **D4a** | Have methodological uncertainties been  addressed by running alternative versions of the  model with different methodological assumptions? | YES |  |
| **D4b** | Is there evidence that structural uncertainties have been addressed via sensitivity analysis? | YES |  |
| **D4c** | Has heterogeneity been dealt with by running model separately for different sub-groups? | YES |  |
| **D4d** | Are the methods of assessment of parameter uncertainty appropriate? | YES |  |
|  | If data are incorporated as point estimates, the ranges used for sensitivity analysis stated  clearly and justified? | YES |  |
| **Consistency (C)** | | | |
| **C1** | Is there evidence that the mathematical logic of the model has been tested thoroughly before  use? | YES |  |
| **C2** | Are the conclusions valid given the data presented? | YES |  |
|  | Are any counterintuitive results from the model  explained and justified? | YES |  |
|  | If the model has been calibrated against independent data, have any differences been  explained and justified? | N/A |  |
|  | Have the results of the model been compared with those of previous models and any differences in results explained? | YES |  |

| **Study: Sau et al. Value in Health 2011: 14 (3); A128.** | | | |
| --- | --- | --- | --- |
| **Quality**  **criteria** | **Question(s) for critical appraisal** | **Response** | **Comments** |
| **Structure (S)** | | | |
| **S1** | Is there a clear statement of the decision problem? | YES | To assess the cost-effectiveness of limited fasciectomy (LF), percutaneous  needle fasciotomy (PNF), and collagenase clostridium histolyticum (CCH) for  the treatment of Dupuytren’s contracture |
|  | Is the objective of the evaluation and model specified and consistent with the stated decision  problem? | YES |  |
|  | Is the primary decision maker specified? | NO | None stated |
| **S2** | Is the perspective of the model stated clearly? | YES | It’s the US healthcare payer perspective |
|  | Are the model inputs consistent with the stated  perspective? | ? | Cannot say since information are not provided in the poster |
|  | Has the scope of the model been stated and justified? | ? | Assumptions although stated, have not been explained sufficiently in the context of available evidence |
|  | Are the outcomes of the model consistent with  the perspective, scope and overall objective of  the model? | YES | The outcomes are consistent with the objective of the model and the perspective of the analysis |
| **S3** | Has the evidence regarding the model structure been described?  Is the structure of the model consistent with a coherent theory of the health condition under evaluation? | ? |  |
|  | Are the sources of data used to develop the structure of the model specified? |  |  |
|  | Are the causal relationships described by the model structure justified appropriately? | NO |  |
| **S4** | Are the structural assumptions transparent and justified? | ? | Assumption are clearly stated but not justified enough |
|  | Are the structural assumptions reasonable given  the overall objective, perspective and scope of the model? | ? |  |
| **S5** | Is there a clear definition of the options under  evaluation? | YES |  |
|  | Have all feasible and practical options been evaluated? | NO | The markov model has been focused only on 3 treatments |
|  | Is there justification for the exclusion of feasible options? | NO | Not provided |
| **S6** | Is the chosen model type appropriate given the decision problem and specified causal  relationships within the model? | YES |  |
| **S7** | Is the time horizon of the model sufficient to reflect all important differences between options? | ? | The model has taken a 10 year life horizon, it’s not clear whether it’s long enough to reflect all important differences |
|  | Is the time horizon of the model, the duration of  treatment and the duration of treatment effect  described and justified? | YES/NO | Treatment duration is not described. No justification is provided for either the time horizon, treatment duration or treatment effect duration |
| **S8** | Do the disease states (state transition model) or the pathways (decision tree model) reflect the underlying biological process of the disease in  question and the impact of interventions? | ? |  |
| **S9** | Is the cycle length defined and justified in terms  of the natural history of disease? | YES/NO | Cycle length has been defined, but not justified |
| **DATA (D)** | | | |
| **D1** | Are the data identification methods transparent and appropriate given the objectives of the  model? | YES |  |
|  | Where choices have been made between data sources, are these justified appropriately? | N/A |  |
|  | Has particular attention been paid to identifying data for the important parameters in the model? | YES |  |
|  | Has the process of selecting key parameters  been justified and systematic methods used to  identify the most appropriate data? | YES | A literature review has been performed to identify and select key parameters |
|  | Has the quality of the data been assessed appropriately? | NO |  |
|  | Where expert opinion has been used, are the  methods described and justified? | N/A |  |
| **D2** | Is the pre-model data analysis methodology  based on justifiable statistical and epidemiological techniques? | NO |  |
| **D2a** | Is the choice of baseline data described and justified? | NO |  |
|  | Are transition probabilities calculated appropriately? | YES |  |
|  | Has a half cycle correction been applied to both  cost and outcome? | YES/NO | Half cycle corrections have been applied, but it’s not clear whether they have been applied to both cost and outcomes |
|  | If not, has this omission been justified? | NO | See above |
| **D2b** | If relative treatment effects have been derived  from trial data, have they been synthesised using  appropriate techniques? | N/A |  |
|  | Have the methods and assumptions used to extrapolate short-term results to final outcomes  been documented and justified? | NO |  |
|  | Have alternative extrapolation assumptions been explored through sensitivity analysis? | NO |  |
|  | Have assumptions regarding the continuing effect  of treatment once treatment is complete been  documented and justified? | NO |  |
|  | Have alternative assumptions regarding the continuing effect of treatment been explored  through sensitivity analysis? | NO |  |
| **D2c** | Are the utilities incorporated into the model  appropriate? | ? | One of the limitation authors mention is that health utility score are extrapolated from patients with carpal tunnel syndrome instead of patients with Dupuytren’s contracture |
|  | Is the source for the utility weights referenced? | YES |  |
|  | Are the methods of derivation for the utility weights justified? | YES |  |
| **D3** | Have all data incorporated into the model been  described and referenced in sufficient detail? | YES/NO | Sources of data have been described but not is great detail due to space constraints (poster) |
|  | Has the use of mutually inconsistent data been  justified (i.e. are assumptions and choices unclear appropriate)? | N/A |  |
|  | Is the process of data incorporation transparent? | YES/NO |  |
|  | If data have been incorporated as distributions,  has the choice of distribution for each parameter  been described and justified? | YES/NO | Parameter distributions are described not justified |
|  | If data have been incorporated as distributions, is  It clear that second order uncertainty is reflected? | NO |  |
| **D4** | Have the four principal types of uncertainty been addressed? | NO | Only parameter uncertainty was performed through one-way sensitivity analysis |
|  | If not, has the omission of particular forms of  uncertainty been justified? | NO | Omission of other forms of uncertainty has not been explained |
| **D4a** | Have methodological uncertainties been  addressed by running alternative versions of the  model with different methodological assumptions? | NO |  |
| **D4b** | Is there evidence that structural uncertainties have been addressed via sensitivity analysis? | NO |  |
| **D4c** | Has heterogeneity been dealt with by running model separately for different sub-groups? | NO |  |
| **D4d** | Are the methods of assessment of parameter uncertainty appropriate? | YES |  |
|  | If data are incorporated as point estimates, the ranges used for sensitivity analysis stated  clearly and justified? | N/A |  |
| **Consistency (C)** | | | |
| **C1** | Is there evidence that the mathematical logic of the model has been tested thoroughly before  use? | NO | No consistency checks are reported |
| **C2** | Are the conclusions valid given the data presented? | YES | Model conclusions are explicable |
|  | Are any counterintuitive results from the model  explained and justified? | N/A |  |
|  | If the model has been calibrated against independent data, have any differences been  explained and justified? | N/A |  |
|  | Have the results of the model been compared with those of previous models and any differences in results explained? | NO | No comparison to previous models have been made |
